# Psychological correlates of hearing protective behaviors in adolescents and young adults: a systematic review

**DOI:** 10.1080/21642850.2025.2507264

**Published:** 2025-06-17

**Authors:** Tjeerd Idger de Zeeuw, Gjalt-Jorn Peters, Lisanne de Regt, Anneloes Baan, Catherine A.W. Bolman

**Affiliations:** a Open University of the Netherlands, Heerlen, The Netherlands; b VeiligheidNL, Amsterdam, The Netherlands

**Keywords:** Behavior change, health prevention, hearing protection, youth, systematic review, psychological determinant, taxonomy

## Abstract

**Background:**

Noise-induced hearing loss is both irreversible and preventable. However, only a minority of adolescents and young adults engage in hearing protective behaviors (HPBs) that reduce their exposure to noise, such as wearing earplugs at music venues. To promote HPBs it is imperative to know the most influential, and potentially modifiable, psychological factors that in this age group stimulate or hinder these protective behaviors.

**Objective:**

The present study aims to offer a systematic literature overview of psychological correlates of HPBs in persons aged 12 to 25 years, and identify the correlates with most potential as behavioral intervention targets.

**Conclusions:**

A total of 82 studies were included in the present review, of which data of 24 studies could be used to assess the strength of the association between one or more psychological factors and HPB. Heterogeneity between studies hindered synthesis. In particular, psychological constructs and HPBs were rarely defined and measured in a uniform manner. Studies were further characterized by a lack of research on psychological factors related to other HPBs than earplug use, such as sound volume control (e.g. the use of a volume limiter). Due to this relative absence of data, associations could not be assessed for specific HPB, and HPBs were aggregated in one variable. Taking into account both the strength of their associations with HPB and their univariate distributions, five psychological factors possessed the greatest potential as behavioral intervention targets: attitude toward recreational noise, perceived barriers, capacity, perceived norms, and perceived threat susceptibility. These results can help the development of new interventions. Additional research is however needed.

## Introduction

### Noise-induced hearing loss

The choices we make, or fail to make, during our youth can have important consequences for the rest of our lives. Prolonged exposure to high levels of noise, or short bursts of very high sound pressure levels, may cause acoustic trauma and, consequently, hearing loss and other hearing problems such as tinnitus (Ding et al., 2019; Lee & Kim, 2018). Once noise-induced hearing loss (NIHL) has occurred, it is irreversible (Ding et al., 2019). NIHL, in addition, may exert an effect on hearing long after harmful noise exposure, as NIHL is associated with greater age-related hearing loss (presbycusis) (De Maria et al., 2020; Moore, 2021). NIHL incurred during adolescence or early adulthood may thus have far-reaching consequences for a person’s quality of life in the years to come. Specifically, hearing loss is known to adversely affect verbal communication and hence hinder social interactions, frequently leading to feelings of exclusion and loneliness (Nordvik et al., 2018; Shukla et al., 2020), and adults with hearing loss are at increased risk for depression (Li et al., 2014). A hearing handicap may, furthermore, thwart or considerably restrict a person’s educational and professional development options, with lower income and decreased employment opportunities as potential ramifications (Emmett & Francis, 2015; Fitzpatrick et al., 2019; Shan et al., 2020).

### Recreational noise exposure in adolescence and early adulthood

It is in adolescence and early adulthood that exposure to recreational noise rises sharply, and often reaches a life-time peak (Beach, Williams, et al., 2013; Williams et al., 2010). Recreational noise, in contrast to noise in occupational settings, is actively sought out for pleasure. In young people, in particular, listening to music on personal listening devices (PLDs) and attending amplified music venues, such as nightclubs and festivals, are popular pastimes. A large portion of young people’s exposure to potentially hazardous levels of noise is during these activities, not only during this particular stage of life, but also with regard to their whole-of-life noise exposure (Beach et al., 2014; Beach, Williams, et al., 2013; Feder et al., 2021; Williams et al., 2010).

Compared to other age groups, a large proportion of teenagers and young adults use PLDs at volumes and durations which may result in hearing deficits (Feder et al., 2021). A systematic review estimated that over 50% of adolescents and young adults, by their use of PLDs alone, exceed the recommended maximum daily noise dose (Jiang et al., 2016). This same, relatively large, group of high risk PLD users is also more likely to be engaged in other loud leisure noise activities (Beach, Williams, et al., 2013; Feder et al., 2021), of which regular concert or nightclub attendance, in particular, contribute to annual noise dose exceedances (Beach, Williams, et al., 2013).

### Exposure to multiple sources of noise

As a consequence of their exposure to multiple sources of loud leisure noise, a considerable portion of teenagers and young adults is exposed to cumulative large levels of noise. Over the whole life cycle, the exposure during adolescence and young adulthood to leisure noise alone may constitute over 50% of the total life-time acceptable noise exposure (Williams et al., 2010), predisposing those most exposed to experience tinnitus (Lee & Kim, 2018) and accelerated or early onset hearing loss. This may, in particular, apply to the group of teenagers and young adults that are also confronted with high levels of occupational noise, now or later in life (Beach, Gilliver, et al., 2013; Neitzel et al., 2014). It is, however, still topic of debate to what extent adolescent leisure noise adversely affects hearing thresholds (Carter et al., 2014; Engdahl et al., 2020; Hoffman et al., 2019).

### Hearing protective behaviors in adolescents and young adults

NIHL is both irreversible and preventable (Rabinowitz et al., 2006). Hearing protective behaviors (HPBs) that reduce the exposure to noise, such as wearing hearing protection (HP) and the use of a volume limiter on a PLD, thus play an important role in long-term hearing health. A minority of teenagers and young adults do, however, engage, in those protective behaviors. A survey among 130,000 Dutch clubbers showed that only 4% used earplugs (Gorter, 2012). Although prevalence rates may vary, in general, the proportion of club, concert and festival visitors that wear HP is less than one in four (e.g. Balanay & Kearney, 2015; Widén, 2013). This means a considerable number of teenagers and young adults expose themselves to potentially hazardous levels of noise, as the volume levels at these music venues on average is about 100 dB, and these venues are frequently attended for several hours per week (Beach et al., 2014). This equals an exposure to noise ratio well above the maximum unprotected exposure levels set for the workplace by the jurisdictions in most Western countries (European Parliament and the Council of the European Union, 2003).

### Psychological factors and hearing protection

While most countries have occupational health and safety laws that oblige the employer to protect workers from occupational noise, in these same countries no or almost no regulations exist with regard to recreational noise (Beach et al., 2020). This means that, outside the workplace, hearing protection is predominantly a personal behavioral choice. For example, it is up to the individual music festival visitor to wear earplugs, yes or no. Similarly, it is up to the individual listener to decide at what sound level and for how long music is played on a PLD. Compared to occupational settings, HPB in in leisure time therefore is to a large extent determined by psychological factors such as individual beliefs and moods.

### Behavior change interventions to increase HPB

In absence of recreational noise jurisdictions, behavior change interventions are likely the most important instrument to increase HPBs in recreational settings. In spite of their potential crucial role, hearing protection interventions leave much to be desired. Generally, systematic and evidence-based approaches in the intervention design are lacking, and the intervention’s main or sole behavior change technique is education, while the effectiveness of the intervention or educational program is not assessed. If the effectiveness is assessed, the effect study is of poor quality, and an effect of the intervention or program on HPB is absent, small, or, at best, moderate (Bramati et al., 2024; Loughran et al., 2020).

### Identifying the most important psychological factors

Well-designed interventions to increase HPBs in adolescents and young adults are therefore highly needed. To enable the systematic design of evidence-based behavioral interventions, the most influential and potentially modifiable psychological determinants of the health behavior in question should be identified (Peters, 2014). To this end, a psychological factor’s association with the health behavior and its univariate distribution need to be considered (Crutzen & Peters, 2022). For factors that theoretically (partially) cause a target behavior, strong associations can evidence causal relevance, and the univariate distribution is important because it shows how much room for improvement there is, e.g. if capacity is strongly correlated with earplug use in students, but the capacity to correctly use earplugs is on average perceived as low by students, then capacity is a potentially promising intervention target as there is room for improvement.

Only after identification of the most relevant, and potentially modifiable, psychological determinants the appropriate corresponding behavioral change principles and their applications can be selected, which form the heart of the intervention. A framework such as the intervention mapping framework (Bartholomew et al., 2016) and a tool to visualize the active components and causal-structural chains of the intervention, such as acyclic behavior change diagrams (ABCDs) (Metz et al., 2022), may help guide this process.

### Aims of the present study

Knowing the most relevant, and potentially modifiable, psychological factors that stimulate or hinder protective behaviors is imperative for developing effective interventions to promote ear-protective behavior in adolescents and young adults. Several studies exist on determinants of hearing protective behavior in this population. However, a review article comprising the most important results of these individual studies is lacking to date. The present study, therefore, aims to offer a systematic literature overview of psychological correlates of HPBs in adolescents and young adults aged 12 to 25 years, and identify those correlates that are likely to be the most promising behavioral intervention targets in this age group.

## Methods

### Pre-registration, protocol, and repository

The Preferred Reporting Items for Systematic reviews and Meta-Analyses (PRISMA) guidelines were followed throughout this article (Moher et al., 2009). Before initiation of the current study, it was pre-registered at the Open Science Framework (OSF) using a registration form for systematic reviews (van den Akker et al., 2023) and PROSPERO websites: https://osf.io/v5jb8 and https://www.crd.york.ac.uk/PROSPERO/display_record.php?RecordID=221758 All materials, including the study protocol, are publicly available at the OSF repository at https://osf.io/53wyk/.

### Literature search

#### Databases and interfaces searched

A systematic literature search was conducted by researcher TZ in the ERIC, PsycINFO, PsycARTICLES, and CINAHL databases with Ebscohost as the interface of choice, and PubMed and Embase databases with, respectively, Pubmed and OVID as interface.

Additionally, grey literature was sought by directly contacting municipal health services in the Netherlands, and by searching websites of Dutch organizations that conduct or report research related to hearing health. Furthermore, the OpenGrey, OAIster, and BASE bibliographic databases were searched for grey literature.

#### Search query

An extensive search query consisting of synonyms and terms related to HPBs (e.g. earplug, volume restriction) and psychological determinants (e.g. attitude, risk perception) was created to search selected bibliographic databases up to June 1st 2021. The specific queries used for each database are available in the OSF repository.

Additional studies were identified by inspecting reference lists of included articles (ascendancy approach) and the articles included in previous systematic reviews on related topics (descendancy approach).

#### Exclusion criteria

A study was excluded if it was not published in English or Dutch, did not involve human subjects, did not report empirical data, did not report on HPBs or proxies thereof (see section ‘HPBs’ for the five HPBs distinguished), or did not report on psychological determinants of HPBs. Studies were additionally excluded if, during data extraction, they turned out to consist of participants with hearing problems only, if the average age of study participants was higher than 30 years or lower than 12 years of age, or if the article reported qualitative data exclusively. A study’s setting (leisure, work, or otherwise) was not an exclusion criterion.

### Selection procedure

#### Eligibility criteria: title, abstract, full article

All articles found in databases, or tracked down by other means, were deduplicated in R, version 4.1.0 (R Core Team, 2022), and imported in reference manager program Jabref (version 2.11.1). In Jabref, articles were inspected based on titles and abstracts by two independent screeners (TZ and AB) in one round, using the study’s exclusion criteria. Text fields for journal names, authors, and publication year were masked from the screeners. An article that was included by at least one of the two screeners proceeded to the subsequent data extraction stage of the review process.

### Data extraction

A standardized extraction script used in conjunction with the ‘metabefor’ package for R (Peters, 2022) was used by researcher TZ to extract the predefined entities from each included study and read into R for later synthesis of the results. All extraction scripts are available in the OSF repository: https://osf.io/53wyk/. During data extraction, articles were fully read. A second reviewer (AB) was consulted if a publication was suspected to be erroneously included. If the two reviewers reached agreement, the study was in- or excluded. A third reviewer (GJP) could be consulted in case of differing opinions or uncertainty.

#### Contacting authors

Authors were contacted through email if information or additional data was required. In most cases an author was contacted with the request to report bivariate associations or other effect size that could be transformed into a correlation, as the estimates published in articles frequently could not be used in the meta-analysis.

#### Extracted variables

An overview of all entities extracted with the standardized extraction scripts can be found at the OSF repository (https://osf.io/s9m7g), and included the following variables:

#### Setting

Adolescents and young adults can be exposed to noise in different settings. In each setting the predominant hearing protection behaviors, and the psychological constructs associated with these behaviors, may differ. In the current study, ‘leisure’, ‘work’, ‘both’, and ‘not specified’ were distinguished.

#### Hearing protective behaviors

‘HPB’ encompasses a range of potential behaviors. In the standardized extraction script the following five behaviors were distinguished: wearing hearing protection (earplugs or earmuffs) when exposed to loud music or noise, restricting the frequency and/or the duration of exposure to loud music or noise, limiting maximum volume when using music players or stereo system, keeping physical distance from sources of loud noise (e.g. speakers at music concerts), and using safe over-ear headphones. In addition, proxies of these behaviors (i.e. intention, willingness, motivation, and action plan) were extracted and classified separately.

#### Psychological constructs

To prevent construct misclassification, constructs were reclassified based on their measurement according to the Decentralized Construct Taxonomy (DCT) (Peters & Crutzen, 2022) developed before the start of the study (available from https://edu.nl/hewyv). The present DCT consists of psychological constructs that according to influential health behavior models and theories (e.g. protection motivation theory, reasoned action approach) or literature may potentially be associated with hearing protection behavior or health behavior in general. In the DCT, each psychological construct received an unequivocal definition, operationalization, and unique construct identifier (UCID).

If fewer than four studies provided data for a psychological construct, if possible, the construct was aggregated with data of a ‘parent’ psychological construct before conducting meta-analyses, for example ‘action regret’ (UCID: https://psycore.one/action_regret_79n2w1bj) was aggregated with the data for ‘anticipated regret’ (UCID: https://psycore.one/anticip_regret_79n2w1bj). The aggregation tree used can be found in the ‘dct-sheets’ folder of the OSF repository.

#### Demographic variables

In addition to HPBs and psychological constructs, a selection of sociodemographic variables were extracted from each study that included age and gender of the participant, socioeconomic status (education, income, or other proxy used), and sample characteristics (‘school students’, ‘university students’, ‘musicians/Djs’, and ‘other’). An overview (as spreadsheet) of the characteristics of the included studies is available in the OSF repository: https://osf.io/53wyk/.

### Meta-analysis

Meta-analyses of univariate results and bivariate associations were conducted with the ‘metafor’ package in R (Viechtbauer, 2010), using random effects models with Hartung-Knapp adjustment. Random effects models were chosen after tests for homogeneity showed the presence of considerable between-study heterogeneity (Q significant at the *p* < .05 level, and *I*^2^ statistic > 50%).

For samples with more than one (homogeneous) result published, before data was entered in the meta-analysis, an average result was composed with metafor’s aggregate function, assuming a dependency of the results of rho = .6.

In circa three quarters of the studies, categorical data series had only two levels (e.g. proportions). Therefore, we converted ordinal results into dichotomous results to enable meta-analyses of proportions with a sufficient number of studies. When ordinal results were converted, ‘sometimes’ counted as ‘no’, and ‘always’ and ‘frequently’ as ‘yes’.

Scores measured on different interval response scales, for the meta-analyses of univariate data, were converted to percent of maximum possible scores (POMP), thus creating relative scores, using the formula 100 * [(observed – minimum) / (maximum – minimum)], where ‘maximum’ and ‘minimum’ refer to the boundaries of the scale, and ‘observed’ refers to the score to convert (Cohen et al., 1999).

For the meta-analyses of associations between psychological constructs and HPBs (or proxies thereof), Pearson's correlations, Spearman's correlations, point-biserial correlations, T-Tests, and Chi-square tests were converted into Fisher’s Z and the corresponding 95% confidence intervals (C.I.). Results were reported as Pearson’s r with 95% C.I. after transformation of the Fisher’s Z estimates.

Authors were contacted if a study reported no effect size for an association, or if a multiple regression model was reported, and asked to report an applicable effect size or to send data to enable the calculation of bivariate associations.

Forest plots were made to graphically display the random-effects models. In the forest plots, squares represent the observed effect sizes or outcomes, and the horizontal line on either side of the square depicts the corresponding 95% C.I. Squares are larger if the estimate is more precise. At the bottom of a forest plot, a four-sided polygon represents the summary estimate. Subsequently summary forest plots were made, which are the plots incorporated in this article. Summary forest plots combine in one plot summary estimates of various meta-analyses, each meta-analysis for a different variable (e.g. different psychological constructs’ associations with HPB). In summary forest plots, each square with horizontal line represent a summary estimates with 95% C.I. More precise estimates have a larger square. The polygon at the bottom of the plot is omitted in summary forest plots.

No risk of bias and quality assessment was performed of included studies, as no such instruments exists for the assessment of so-called ‘determinant studies’, and criteria used by existing instruments were not relevant for the present review.

### Ethical statement

This study has been exempted from ethical review by the ethics committee of the Faculty of Psychology of the Open University, as it does not involve human participants.

## Results

### Study selection

**Figure 1.**
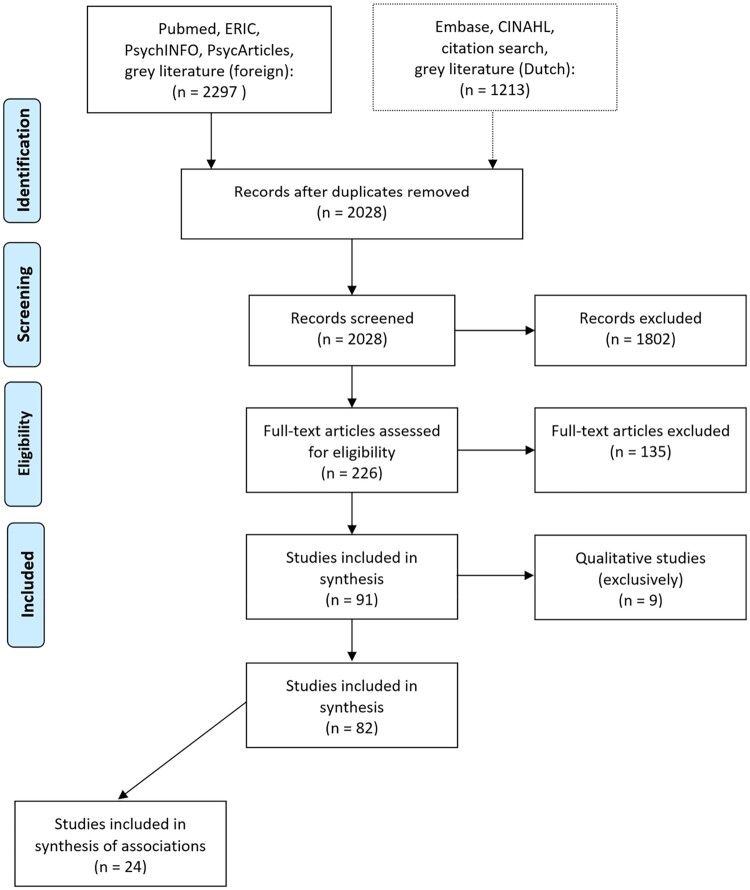
Flow chart of the studies included in the review.

In total, 82 studies were included for synthesis in the systematic review. The majority of studies (*n* = 58, 70.7%) reported univariate analyses only, while 24 studies (29.3%) were eligible for synthesis of a correlation between at least one psychological construct and one HPB (or proxy thereof; see Figure 1).

### Characteristics of the included studies

Supplemental Table S1 shows the characteristics of the 82 studies. Studies were conducted in 17 different countries. The United States (*n* = 25), the Netherlands (*n* = 14), Belgium (*n* = 9), Australia (*n* = 8), and Sweden (*n* = 5) were most prominently represented. Eleven studies were not published in peer reviewed academic journals and, therefore, labeled as ‘grey literature’ (Mahood et al., 2014). Nine of these eleven studies were conducted in the Netherlands. Studies were published from 2003 to 2021 and sample sizes ranged from *n* = 29 (Portnuff et al., 2011) to *n* = 130,000 (Gorter, 2012). Participants were, on average, 20 years of age (*M* = 19.95, for 60 samples with mean age reported).

#### Study participants

High school and university students formed the most prevalent group of study participants: in 41.5% (*n* = 34) and 32.9% (*n* = 27) of the studies the sample was composed predominantly of high school and university students, respectively. In a smaller number of studies, study participants were clubbers/music venue visitors (*n* = 9, 11.0%), musicians (*n* = 5, 6.1%), or workers/employees (*n* = 3, 3.7%). Some studies had an unknown or heterogeneous composition of study participants (*n* = 10, 12.2%), or participants were classified in more than one group (e.g. conservatory students were classified both as university students and musicians).

#### Theories and models used in the studies

Several theories and models were either used as framework in the study or referred to in the publication. The theory most often used as framework was the Health Belief Model (*n* = 12) (Rosenstock, 1974), followed by Widén’s (2013) model of decision-making regarding hearing conservation, and the Transtheoretical Model (‘Stages of Change’) (*n* = 4 each) (Prochaska & DiClemente, 2005). However, the related Theory of Reasoned Action (Fishbein & Ajzen, 1975), Theory of Planned Behavior (Ajzen, 1991) and Reasoned Action Approach (Fishbein & Ajzen, 2010) together were mentioned seven times as framework for a study.

#### Questionnaires used in the studies

The questionnaires most frequently used in the 82 included studies were the Youth Attitudes to Noise Scale (YANS; *n* = 22) (Olsen-Widén, 2004a) and the Beliefs About Hearing Protection and Hearing Loss scale (BAHPHL; *n* = 9) (National Institute for Occupational Safety and Health, 1998), both predominantly in the adapted version by Keppler (2010). The Adolescents’ Habits and Use of Hearing Protection scale (AHH) (Olsen-Widén, 2004a) was a third questionnaire frequently used (*n* = 6). Other instruments that were applied in multiple studies were the Listening Habits Questionnaire (*n* = 4) (Portnuff, 2011), the Personal Listening Device and Hearing Questionnaire (*n* = 3) (Danhauer et al., 2009), and the Hearing Symptom Description scale (HSD; *n* = 3) (Olsen-Widén, 2004b).

#### Design of the included studies

Studies included in the review almost exclusively (96%) were cross-sectional surveys.

### Hearing protective behaviors under study

#### Wearing hearing protection

The use of hearing protection (HP), such as earplugs, was the HPB most often measured in studies (*n* = 54). In the majority of these studies (*n* = 41), past or current *behavior* was measured, i.e. wearing earplugs or earmuffs when exposed to noise. In a minority of studies (*n* = 11) both HP use (i.e. *behavior*) and the *intention* to wear HP were (separately) measured. In three studies the intention to wear HP, but not the actual behavior, was assessed.

#### Hearing protection use measurements

The use of HP was measured in different ways and in different settings. As both of these aspects may have an impact on the prevalence of HP use and the strength of associations between HP use and psychological correlates, HP use was categorized according to measurement and setting.

#### ‘Ever used’ and ‘when exposed to loud noise’

In seven studies (*n* = 4,567), participants were asked whether they had ‘ever used’ HP (yes/no), without specifying a context or timeframe. In 18 studies, the question was asked whether participants used HP ‘when exposed to loud noise’ (*n* = 23,556).

#### The use of hearing protection in leisure settings

In various studies, the use of HP was measured in specific leisure time settings. The following six settings were identified: festival and/or concert (15 studies, *n* = 134,832), disco and/or nightclub (15 studies, *n* = 7,842), when making music (10 studies, *n* = 8,84), when using power tools (7 studies, *n* = 1,575), when using firearms (6 studies, *n* = 911), and during sports practice or sports events (4 studies, *n* = 1,498). Data of settings that were measured in less than four studies were not aggregated (e.g. HP use in cafe/pub).

#### The use of hearing protection in occupational settings

The use of HP in work settings was measured infrequently in this younger age group (5 studies, *n* = 546).

**Figure 2.**
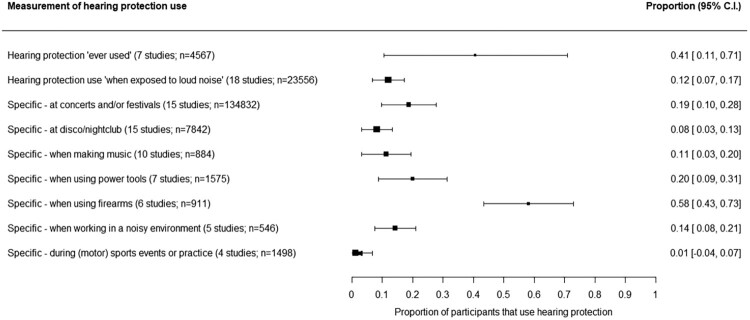
Forest plot of overall prevalence of hearing protection use in various settings.

Figure 2 depicts the prevalence rates for the nine measurements of HP use. The highest prevalence was observed for HP use during firearm use, with on average 58% (95% C.I. 43%–73%) of participants using HP when using a firearm. In turn, HP use during sports practice or sports events was lowest (and non-significant): 1% (95% C.I. −4% to 7%).

#### Choosing a safe sound volume and using a volume limiter

Ten studies assessed HPB (or the intention to engage in that behavior) with regard to the use of PLDs. Six studies assessed participants’ choice for a safe sound volume to protect hearing, and in four studies participants were asked whether they used or intended to use a volume limiter.

#### Other hearing protective behaviors assessed

Limiting the exposure to loud music or noise was a HPB assessed in four studies. Other HPBs (or proxies thereof) assessed were taking ‘noise breaks’ (*n* = 4), keeping distance from speakers (*n* = 2), using safe headphones (*n* = 1), and ‘general’ HPB (*n* = 5). The latter category comprises HPB described in general terms, e.g. ‘I intend to protect my hearing during discotheque visits from now on’ (Vogel et al., 2010).

### Psychological constructs

Psychological constructs measured in the included studies were classified according to the previously developed taxonomy (i.e. the DCT). Construct classification was based on the construct’s operationalization and, if provided, its definition. Therefore, constructs could be classified differently than if they would be classified based on the name provided by the study’s authors, e.g. a study’s measurement of ‘perceived behavioral control’ could be classified as being a reflection of ‘capacity’.

If after classification, fewer than four studies provided data for a psychological construct, the construct was aggregated with (similar) ‘sibling’ psychological constructs into a ‘parent’ construct, following the structure of the aggregation tree set up before the start of data synthesis. If a construct could not be aggregated into a ‘parent’ construct, the results were used unaggregated.

#### Aggregation of HPBs for the meta-analyses of associations

In the 24 studies eligible for synthesis of correlations between psychological constructs and a HPB, the HPB in question was earplug use (*n* = 22), restrict sounds volume during PLD use (*n* = 2), limit exposure to recreational noise (*n* = 2), and taking steps to protect hearing (*n* = 1). For the meta-analyses of associations, all HPBs (earplug use, safe volume choices, limit exposure, and the generic measure) were aggregated, and associations between psychological constructs and this aggregate of HPB were calculated. It was not possible to conduct meta-analyses for the separate HPB, due to the limited number of available studies. For the intention to engage in HPB, the same method was used; all HPBs were again aggregated and meta-analyses for the associations between this aggregate and psychological constructs were performed.

### Univariate results for potential psychological correlates of hearing protective behavior

For the meta-analysis of univariate results, POMP scores, means, and proportions were pooled separately.

#### POMP-scores for 12 psychological constructs measured with different questionnaires

Scores for psychological constructs grouped in the same DCT-category but measured on different interval response scales were converted to POMP scores, with 0 and 100 being respectively the minimum and maximum possible score, with a higher score being indicative of a greater presence of hearing protective beliefs and/or behavior.

**Figure 3.**
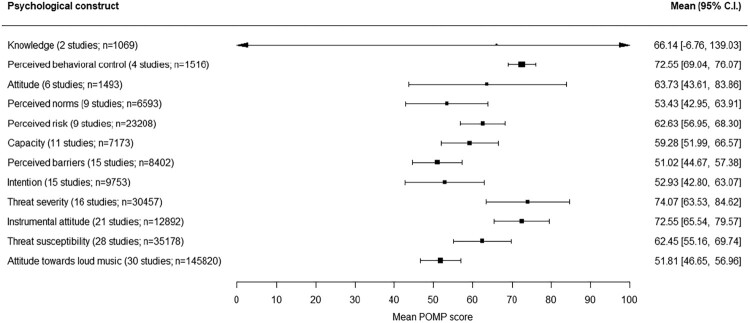
Mean POMP scores and 95% C.I. for 12 psychological constructs measured with differing questionnaires.

Twelve psychological constructs were measured in more than one study and were therefore taken into account in the univariate analyses. As can be seen in Figure 3, Perceived behavioral control was the construct with the highest score (*M* = 72.6, 95% C.I. 69.0–76.1), meaning that study participants on average assume that they are capable of, and have control over, protecting their hearing against noise. The score for attitude towards loud music was the lowest (*M* = 51.8, 95% C.I. 46.6–57.0) meaning that study participants on average had a neutral attitude towards loud music.

#### Mean scores for nine psychological constructs measured with the YANS and BAHPHL

The results for the YANS (Olsen-Widén, 2004a) and the BAHPHL (National Institute for Occupational Safety and Health, 1998) are part of the previous meta-analysis of POMP scores presented above. In addition, a separate meta-analysis was performed with exclusively data of the YANS and BAHPL. Only these two questionnaires were used in enough studies (*n* = 22 and *n* = 9, respectively) to enable pooling of unconverted scores. For nine psychological constructs measured with the subscales of the YANS and the BAHPHL, mean scores and 95% C.I. were calculated.

**Figure 4.**
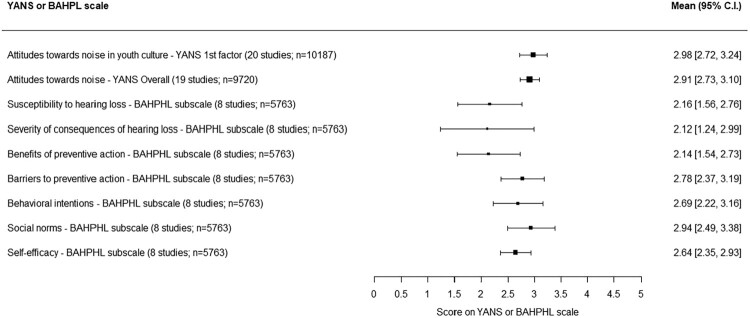
Mean scores and 95% C.I. for psychological constructs measured with subscales of the YANS and BAHPHL.

The YANS and BAHPL consist of five-point Likert scales with ‘totally agree’ (coded as 1) and ‘totally disagree’ (coded as 5) at the utmost poles. For the meta-analysis, scores were reversed so that a higher score indicates a greater presence of hearing protective beliefs and/or behavior. The (unconverted) univariate results for the YANS and BAHPL are displayed in Figure 4.

As can be observed in Figure 4, the ‘attitudes towards noise associated with elements of youth culture’ of the YANS was the subscale most often used (*n* = 20). No results stand out: all psychological constructs have a mean score close to 2.5 and the 95% C.I. of the constructs overlap, meaning mutual differences are not significant.

#### Proportion of participants that agree with statements

Psychological determinants of a behavior are made up of subdeterminants, that can be defined as ‘determinants at a lower level of psychological generality that are theoretically assumed to predict or be a part of overarching determinants’ (Peters & Crutzen, 2018). For example the belief ‘Earplugs are uncomfortable’ is part of the determinant perceived barriers (to wear earplugs). Knowing these subdeterminants may help to select the specific aspects of a psychological determinant that should be targeted in a behavioral intervention.

**Figure 5.**
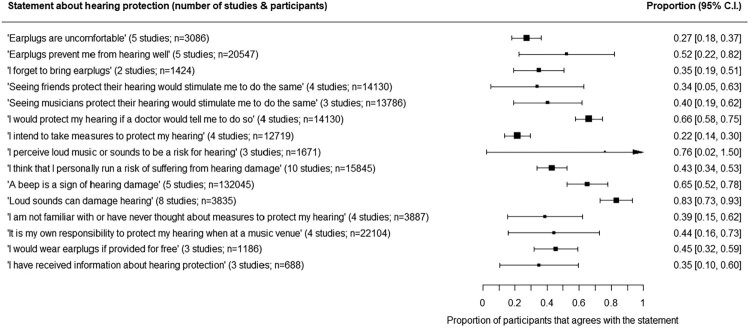
Number of participants (as frequencies) that agree with frequently used statements about hearing protection.

Fifteen statements, in comparable wordings, were used in various studies to measure different subdeterminants of hearing protective behaviors. Agree or disagreement with the statements was measured on a nominal scale or converted from a ordinal to nominal scale. The number of participants (as frequencies) that concur with each statement are shown in Figure 5.

The statement with which participants agreed the least (*p^* = 0.22, 95% C.I. 0.14–0.30) was ‘I intend to take measures to protect my hearing’. The statement was measured in four studies. In contrast, ‘Loud sounds can damage hearing’, included in eight studies, was the statement most participants agreed on (*p^* = 0.83, 95% C.I. 0.73–0.93). Furthermore, about a three quarters of participants did *not* agree with the statement ‘Earplugs are uncomfortable’, measured in five studies. Finally, sixty six percent of the participants (95% C.I. 0.58–0.75) agreed with the statement ‘I would protect my hearing if a doctor would tell me to do so’, that was part of four studies.

### Associations between psychological factors and hearing protective behaviors

The linear associations between psychological constructs and, respectively, hearing protective behavior (HPB), and the intention to engage in HPB, were separately pooled. Associations for perceived norms were only measured in included studies in relation to the use of hearing protection, or the intention to use hearing protection, and not in relation to other HPBs. The same applied to perceived barriers, with the exception of one study that measured its association with noise exposure avoidance.

#### Associations with the intention to engage in HPB

**Figure 6.**
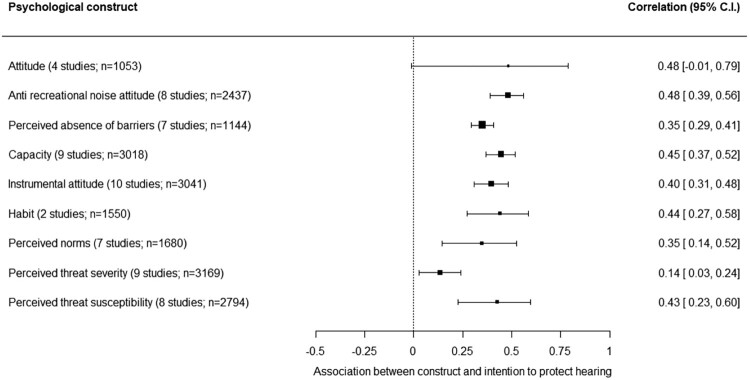
Associations between nine psychological constructs and intention to engage in hearing protective behavior (HPB).

The associations of nine, alphabetically ordered, psychological constructs with the intention to engage in HPB are shown in Figure 6. The association for attitude is not significant (the 95% C.I. crosses zero). With the exception of the association for ‘perceived threat severity’ (*r* = .14), the strength of the associations is from *r* = .35 to *r* = .48, which is considered weak to moderately strong (Schober et al., 2018), with overlapping 95% C.I.s, meaning the strength of the associations of these six psychological constructs do not significantly differ from each other.

#### Associations with HPB

**Figure 7.**
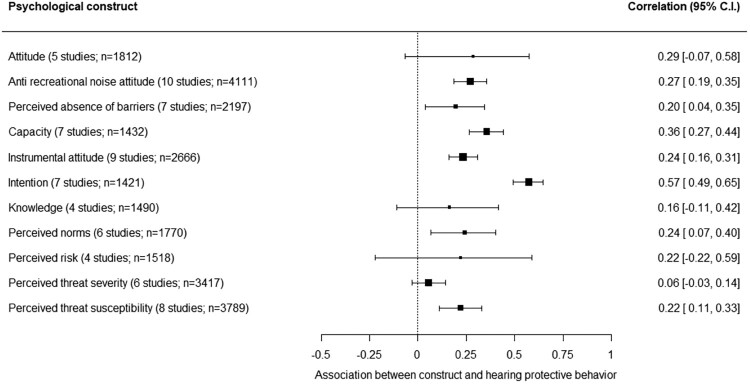
Associations between 11 psychological constructs and hearing protective behavior (HPB).

The associations of 11, alphabetically ordered, psychological constructs with HPB are shown in Figure 7. The associations for attitude, knowledge, perceived risk, and perceived threat severity are not significant (the 95% C.I. of these correlations cross zero). The associations for the remaining seven psychological constructs are from *r* = .20 to *r* = .57. Only intention’s association with HPB is moderately strong (*r* = .57, 95% C.I. *r* = .49 – *r* = .65). The strength of the other associations is weak (Schober et al., 2018). Only intention’s 95% confidence interval does not overlap with the confidence interval of other constructs significantly associated with HPB. Intention’s association with HPB is, thus, significantly stronger compared with the other constructs. The other six constructs’ associations with HPB do not differ significantly from each other in strength.

## Discussion

The present study offers a systematic review of psychological factors associated with hearing protective behaviors (HPBs) in adolescents and young adults aged 12 to 25 years and identifies the potentially most promising behavioral intervention targets in this age group.

Participants of the 82 studies included in this review generally agreed that loud sounds can damage hearing, and that they are capable of, and have control over, protecting their hearing against these loud sounds. These pro-hearing protective beliefs are, however, counteracted by a neutral attitude towards loud music and a low to moderate intention to take hearing protective measures. This is reflected by adolescents and young adults’ actual behavior: only a minority of them take measures to protect their hearing against high sound volumes. Of those that go clubbing or attend a live music gig, activities that expose many young people to potentially harmful noise levels every weekend, respectively 8 and 15 percent wears earplugs, the studies included in this review show.

To help explain and potentially change behavior, data of 24 studies was used to identify the psychological factors most strongly associated with hearing protective behavior (HPB). Attitude toward recreational noise, perceived barriers, capacity, instrumental attitude, perceived norms, and perceived threat susceptibility^1^ were found to be weak to moderately associated with the intention to engage in HPB, and weakly associated with HPB itself. They did not differ significantly in the strength of their associations. Intention was the sole psychological factor that showed a moderate association with HPB. Attitude, knowledge, perceived threat severity, and perceived risk were found not to be significantly associated with HPB or the intention to engage in HPB.

Besides the strength of the association with the behavior of interest, a psychological construct’s univariate distribution offers important information about its potential as a behavioral intervention target, as it reflects the room for improvement (Crutzen & Peters, 2022). Taking into account both the strength of the association and the univariate distribution, instrumental attitude appeared to be less apt as intervention target, narrowing the list of potentially promising behavioral intervention targets to five: attitude toward recreational noise, perceived barriers, capacity, perceived norms, and perceived threat susceptibility.

In the present study, intention had the strongest association with HPB of all psychological constructs, and its univariate distribution suggests there is room to improve intention in a considerable part of the adolescents and young adults. However, it is not considered a suitable intervention target by behavior change theorists and interventionists, as intention is hard to change directly but rather is increased through a change in its causal antecedents (e.g. capacity or perceived norms) (Morwitz & Munz, 2021).

Important similarities and differences in the outcomes of the current study can be observed with respect to previous works. However, different methodologies make comparison possible only to a limited extent. Importantly, in contrast to two important studies on psychosocial correlates of HPB in young people by Vogel et al. (2007) and Widén (2013), no single theoretical framework was used in the current study to help categorize psychological correlates. Instead, the DCT set up before the start of the study guided this process. Moreover, the present study identified psychosocial correlates of ‘HPB’, which encompassed several HPBs, including earplug use and safe sound volume choices, an approach similar to the method used by Vogel et al. (2007), and unlike research on psychosocial correlates of one specific hearing protective behavior, e.g. earplug use, as in Widén (2013).

Notwithstanding methodological differences, across studies a common image appears on which psychosocial factors are influential, and which are not, in stimulating hearing protective behavior in young people. Firstly, information provision with the aim to increase knowledge, and stressing the severity of hearing damage, are not likely to change behavior. In interventions to increase HPB in young people, these are the most commonly used, and often only, behavior change methods (Loughran et al., 2020). This study, and numerous previous studies, suggest that this will not change behavior. Not surprisingly, interventions to increase HPB in young people are usually not very effective (Khan et al., 2018; Loughran et al., 2020).

What is more likely to encourage behavior change, the current study shows, is to make young people aware of their personal vulnerability. If young people acknowledge or experience that their behavior has negative personal consequences, they are more willing to change it. However, hearing problems often only becomes noticeable in the long term, while young people are especially sensitive to short-term consequences, such as the immediate feeling of joy loud music may give. Although this was not investigated in the present study, it therefore seems useful to specifically emphasize possible short-term negative consequences of their behavior to young people, such as developing permanent tinnitus (Bruijn de et al., 2016; Hall et al., 2015).

In line with Vogel et al. (2007), Widén (2013), and other previous research, the present study also shows that interventionists should aim to increase young people’s capacity to change their behavior. Inducing fear and a sense of vulnerability, will more likely change behavior if a person possesses the capacity required to change (ten Hoor et al., 2025). This can involve teaching practical skills, such as learning someone to insert earplugs or turn on a volume limiter, but can also involve increasing self-control in order to resist the impulse to choose unsafe sounds volumes.

An important aspect that cannot be ignored when one intends to increase hearing protective behavior, is that loud sounds, specifically loud music, in many people, young and old, elicits a strong pleasant effect (Welch & Fremaux, 2017). The present study shows that when young people have a more positive attitude toward recreational noise (e.g. music played at high volume in clubs), they are less likely to engage in HPB. In addition, the present study shows most adolescents and young adults know loud noise can harm their hearing, and they are aware of hearing protection measures, yet they do not take these measures. Vogel et al. (2007) explains this apparent contradiction by applying the protection motivation theory (Rogers, 1975): ‘the probability of an adaptive response is decreased by the perceived rewards of a maladaptive response’, that is; the expected joy loud music will give outweighs the possible negative consequences it may have. Interventionists can take this information into account by endorsing, as much as possible, hearing protective measures that do not reduce music’s pleasant effect. Examples of such measures are promoting the use of headphones with noise canceling, and stimulating the use of higher quality earplugs with music filter.

Evident from this review, as in the review by Vogel et al. (2007), is that barriers may prevent young people from engaging in HPBs. These perceived barriers may relate to disadvantages associated with wearing earplugs, such as appearance or discomfort. In Widén’s study (2013), perceived barriers was the variable that, together with norms, was most strongly associated with the wearing of earplugs. These barriers are thus not trivial, but are to be taken seriously and should be addressed in interventions. With the exception of one study, studies included in this review measured the association between barriers and the use of hearing protection. However, barriers can potentially also influence safe listening practices; e.g. a youngster may regard choosing a high volume unavoidable when using headphones in the presence of ambient noise. However, whether the presence of perceived barriers is a relevant determinant of choosing a safe volume could not be assessed in the present study.

Adolescence and young adulthood, in particular, are life stages in which behavior is influenced by beliefs about peers’ behavior and approval (Baumgartner et al., 2011). In line with these findings, the present review demonstrates that social norms influence young people’s use of earplugs. These social norms can be divided into injunctive and descriptive norms (Fishbein & Ajzen, 2010); i.e. respectively young people’s perception that their friends (or other important others) think they should wear or should not wear earplugs, and young people’s perceptions that their friends are or are not wearing earplugs. Due to an absence of sufficient data, it was not possible to determine whether the first or the latter perception is more influential. Neither could be determined whether social norms play a role with regard to other HPBs than the use of hearing protection, as this was not assessed in the included studies. However, social norms are most influential with regards to behaviors that are visible to others (Yamin et al., 2019) and thus likely have a greater impact on earplug use, which takes place mostly in social settings such as a music festival, than on safe headphone use, which is more an individual and less overt choice.

### Limitations

The current study has a number of limitations. Firstly, studies performed in Western countries on hearing protection in leisure time, not at work, are overrepresented in the review. In addition, the vast majority of studies focus on the use of earplugs, while safe music player use and other leisure time hearing protective behaviors receive far less attention. Study participants for the largest part are high school and university students around twenty years old. Due to these characteristics of included studies, the findings of the present review may not generalize to populations and hearing protective behaviors underrepresented in the included data.

Secondly, significant heterogeneity was observed across studies. Equal to Vogel et al. (2007), this hindered overall assessments of the strength of associations. Psychological constructs and HPBs (and their proxies) were rarely defined and measured in a uniform manner. The operationalization and definition of psychological constructs frequently differed to such a degree between studies that, although the same name was used for a construct (e.g. ‘risk perception’), the latent construct measured was not the same. Comparison and aggregation of study results nonetheless remained, to some degree, possible due to the DCT developed before the start of the review. Notwithstanding, to what extent differences between the psychological constructs’ pooled effect estimates reflect real world and methodological diversity remains difficult to determine. However, the main results of the present study are observed across studies, increasing the likelihood that these findings, most notably the identification of the most influential and potentially modifiable determinants of HPB, do indeed correctly reflect what researchers in this study domain have consistently observed.

An additional limitation of the present study is the use of an aggregate of HPB for the meta-analyses of associations. An absence of sufficient data prevented analyses for each type of HPB separately. By joining all HPBs, important information may not have shown up, as the importance of a psychological construct for the engagement in the behavior potentially depends on the specific HPB and setting at hand. Similarly, aggregating ‘sibling’ psychological constructs into ‘parent’ constructs, when less than four studies reported results for the ‘sibling’ construct, may have introduced a loss of information. This loss did not occur when a construct was not aggregated (if similar results were absent). However, meta-analysis with fewer than four studies, increases the unreliability of the outcomes (Fu et al., 2011).

Finally, the reader should take into account that the present review does not include articles that exclusively report non-quantitative results and/or study non-linear relationships between psychological factors and HPB, and that bibliographic databases were searched up to June 1st 2021. This may have influenced the selection of studies included in this review.

### Strengths

The current review is based on an extensive literature search, including grey literature, in which various databases were consulted. Another strength of the study is the use of a Decentralized Construct Taxonomy (DCT) (Peters & Crutzen, 2022) developed before the start of the review (https://edu.nl/hewyv) to classify the psychological constructs measured in the included studies. This reference to unequivocal definitions and operationalizations, to some degree, made comparison and aggregation of study results possible, despite the great heterogeneity in which psychological constructs were defined and measured in studies. The DCT, moreover, may help researchers and intervention developers give a clearer idea what they need to measure or change when they set up a new study or behavioral intervention (Peters & Crutzen, 2024).

## Conclusion

This review identifies five psychological factors as promising intervention targets to increase hearing protective behaviors in adolescents and young adults: attitude toward recreational noise, perceived barriers, capacity, perceived norms, and perceived threat susceptibility. These findings may help guide the development of behavioral interventions for this age group. Additional research is however needed to further improve such interventions; little is known about the psychological factors that drive other hearing protective behaviors than earplug use and, in young people, research on hearing protective behaviors at work and in non-Western countries is underrepresented. Our understanding of psychological factors underlying hearing protective behavior is however most helped if in future research clear and more uniform definitions and operationalizations are used. This would greatly help improve research data comparison and aggregation.

## Supplementary Material

Supplemental_material_Table_S1.docx

## Data Availability

All materials are publicly available at the study’s OSF repository at https://osf.io/53wyk/.

## Note

1 For definitions and operationalizations of the psychological constructs, see the DCT at: https://edu.nl/hewyv.

## Open Scholarship

This article has earned the Center for Open Science badges for Open Data, Open Materials and Preregistered. The data and materials are openly accessible at , and .
