# Supplementary material for: Psychological correlates of hearing protective behaviors in adolescents and young adults: a systematic review
Source: Health Psychol Behav Med. 2025 Jun 17;13(1):2507264. doi: 10.1080/21642850.2025.2507264 (PMC12175190; doi:10.1080/21642850.2025.2507264)
Supplement: Supplemental_material_Table_S1.docx [file RHPB_A_2507264_SM0360.docx]

Table 1. Characteristics of the included studies.

| **Author** | **Title** | **Year** | **Country** | **N** | **Participants** | | | **Setting** | **Psychological construct**  ^(DCT UCID)^ | **Theory** | **Instrument** | | | **Hearing protection behavior** | | | **Association** | **Grey lit.** |
| --- | --- | --- | --- | --- | --- | --- | --- | --- | --- | --- | --- | --- | --- | --- | --- | --- | --- | --- |
|  |  |  |  |  | **Gender** | **Age** | **Group** |  |  | ^R=reference; F=framework^ | **YANS** | **BAHPL** | **Other** | **Earplug use** | **PLD use** | **Measurement** |  |  |
| Alnuman, N., & Ghnimat, T. | _Awareness of Noise-Induced Hearing Loss and Use of Hearing Protection among Young Adults in Jordan_ | 2019 | JO | 245 | M=53.9%  F=46.1% | M=21.5 SD=2.2 | U | Leisure  ‘Noisy environment’ | _behaviour_79n2w1bj_  _inflEnvironment_79n2r0sy_  _knowledge_79n2fh4b_ | 0 |  |  |  | X |  | 1) Behavior:  HP use (*‘ever worn’*)  (yes/no) |  |  |
| Ameye et al. | _Survey of Recreational Noise Exposure through the Use of Personal Music Players in Young Nigerian Adults_ | 2016 | NG | 985 | M=60.3%  F=39.7% | M=24.4  SD=6.0 | U | Leisure | _behaviour_79n2w1bj knowledge_79n2fh4b_  _risk_perception_79n2fh4t threat_susceptibility_79n2fh4s_ | 0 |  |  |  |  |  | LB |  |  |
| Auchter, M., & Le Prell, C. G. | _Hearing loss prevention education using Adopt-a-Band: Changes in self-reported earplug use in two high school marching bands_ | 2014 | US | 93 | M=59.1%  F=40.9% | No age reported  _(all participants are U.S. high school students, i.e. 14-18 years of age)_ | M  S | Leisure | _behaviour_79n2w1bj_  _inflEnvironment_79n2r0sy threat_susceptibility_79n2fh4s_ | HBM (R) |  |  |  | X |  | 1) Behavior: HP use while  at marching band practice (4-point scale) |  |  |
| Balanay, J. A. G., & Kearney, G. D. | _Attitudes toward Noise, Perceived Hearing Symptoms, and Reported Use of Hearing Protection among College Students: Influence of Youth Culture_ | 2016 | US | 2151 | M=34.9%  F=64.6%  O=0.4%  NR=0.1% | M=19.1 SD=1.5 | U | Leisure | _attitude_noise_7c08258d_  _attitude_soundculture_7c082lmy behaviour_79n2w1bj_ | TPB (R) | X |  | AHH   HSD | X |  | 1) Behavior:  HP use (yes/no) for 7 leisure time activities. | X |  |
| Beach, E. F., Nielsen, L., & Gilliver, M. | _Providing earplugs to young adults at risk encourages protective behaviour in music venues_ | 2016 | AU | 51 | M=72.5%  F=27.5% | M=27.1  Min.=27.0  Max.=39.0 | C  U _(>50%)_ | Leisure | _behaviour_79n2w1bj_  _risk_perception_79n2fh4t_ | HBM (R)  TTM (R)  Libertarian paternalism (R) |  |  |  | X |  | 1) Behavior:  HP use at pub, festival, concert, nightclub, and gigs (5-point scale). |  |  |
| Beach, E. F., & Gilliver, M. | _Time to Listen: Most Regular Patrons of Music Venues Prefer Lower Volumes_ | 2019 | AU | 933 | M=56.9%  F= 43.1% | M= 26.2 SD=9.6 | C | Leisure | _attitude_soundculture_7c082lmy_  _intention_73dnt604_  _locus_of_control_7ddfj4wn threat_susceptibility_79n2fh4s_ | CAALM model (R)  Social identity theory (R) |  |  |  | X |  | 1) Behavior:  HP use *if provided for free* at, respectively, nightclubs and life music venues (5-point scale) |  |  |
| Bogoch, I. I., House, R. A., & Kudla, I. | _Perceptions about hearing protection and noise-induced hearing loss of attendees of rock concerts._ | 2005 | CA | 204 | M=55.4%  F= 44.6% | M=20.6  Mdn=19.0  Min.=14.0  Max.=65.0  _(94.2% under age 30)_ | C | Leisure | _behaviour_79n2w1bj_  _inflEnvironment_79n2r0sy threat_susceptibility_79n2fh4s_ | TTM (F) |  |  |  | X |  | 1) Behavior:  HP use at concerts (yes/no) |  |  |
| de Bruijn et al. | _Testing the effects of a message framing intervention on intentions towards hearing loss prevention in adolescents_ | 2016 | NL | 375 | M=45.1%  F= 54.9% | M=15.1 SD=1.2 | S | Leisure | _intention_73dnt604_  _risk_perception_79n2fh4t_ | Message framing theory (F)  Construal Level Theory (F)  _for intervention,_ *_not_* _relevant for extracted data)_ |  |  |  |  | X | 1) Intention to listen to music at a reduced volume | X |  |
| Callahan et al. | _Collegiate musicians’ noise exposure and attitudes on hearing protection_ | 2011 | US | 130 | M=53.0%  F=47.0% | M=19.4  Min.=18.0  Max.=25.0 | M  U | Leisure | _autonomy_conditionPower_73dnt5zs_  _behaviour_79n2w1bj threat_susceptibility_79n2fh4s_ | 0 |  |  |  | X |  | 1) Behavior:  HP use during instrument use (yes/no) |  |  |
| Callahan et al. | _Effectiveness of a noise-induced hearing loss seminar for collegiate musicians_ | 2012 | US | 129 | M=52.7%  F=47.3% | M=18.0 SD=25.0 | M  U | Leisure | _emotion_79n2r0sy threat_susceptibility_79n2fh4s_ | 0 |  |  |  |  |  | NC |  |  |
| Carter, L., & Black, D. | _More to Lose? Noise-Risk Perceptions of Young Adults with Hearing Impairment_ | 2017 | AU | 210  _(total sample)_  131  _(group no HI)_ | M=40.5%  F=59.5%  _(group no HI)_ | M=22.4 Min.=18.0  Max.=24.0  _(group no HI)_ | _Data of the group with HI (n=79) was_ *_not_* _used in the review._ | Leisure | _behaviour_79n2w1bj risk_perception_79n2fh4t_ | 0 |  |  |  | X |  | 1) Behavior:  HP use for 16 leisure time activities (3-point scale).  _(For the review a selection was made of 6 activities)_ |  |  |
| Chesky et al. | _Attitudes of college music students towards noise in youth culture._ | 2009 | US | 467 | M=60.0%  F=40.0% | M=19.8  SD=3.6 | M  U | Leisure | _attitude_soundculture_7c082lmy intention_73dnt604_ | TPB (F) | X |  |  |  |  | 1) Intention (to prevent exposure to loud sounds (5-point scale) _(i.e. 4th factor of YANS)_ | _Study reports correlation between two subscales of the YANS (therefore, correlation_ *_not_* _used for review)_ |  |
| Chung et al. | _Evaluation of noise-induced hearing loss in young people using a web-based survey technique._ | 2005 | US | 9458 | M=35.0%  F=65.0% | M=19.2 SD=NR |  | Leisure | _autonomy_conditionPresence_73dnt5zr_  _behaviour_79n2w1bj_  _descrNorms_belief_73dnt5zm intention_73dnt604_  _motivationToComply_73dnt5zf perceivedNorms_73dnt5zq risk_perception_79n2fh4t_  _threat_susceptibility_79n2fh4s_ | 0 |  |  |  | X |  | 1) Behavior:  HP use (*‘ever worn’*) at places where loud music is played  (yes/no)    2) Intention to wear HP the next time a concert, rave, or club is attended (4-point scale) |  |  |
| Crandell, C., Mills, T. L., & Gauthier, R. | _Knowledge, Behaviors, and Attitudes About Hearing Loss and Hearing Protection Among Racial/Ethnically Diverse Young Adults_ | 2004 | US | 200 | M=50.0%  F=50.0% | Min.=18.0  Max.=29.0 | U | Leisure  ‘Noisy environment’ | _behaviour_79n2w1bj_  _knowledge_79n2fh4b risk_perception_79n2fh4t_ | HBM (F)  TTM (F) |  |  |  | X |  | 1) Behavior:  HP use when exposed to loud noise (5-point scale) |  |  |
| Danhauer et al. | _Survey of college students on iPod use and hearing health_ | 2009 | US | 609 | M=40.0%  F=60.0% | M= 19.6  _(calculated, frequencies reported)_ | U | Leisure | _awareness_79n2w1bj_  _instrumentalAttitude_73dnt5zb intention_73dnt604_  _knowledge_79n2fh4b risk_perception_79n2fh4t_ | 0 |  |  | PLDHQ |  | X | 1) Intention to use volume restriction (4-point scale) |  |  |
| Danhauer et al. | _Survey of high school students' perceptions about their iPod use, knowledge of hearing health, and need for education_ | 2012 | US | 131 | M= 33.9%  F= 66.1% | M= 17.2  _(calculated, frequencies reported)_ | S | Leisure | _awareness_79n2w1bj_  _instrumentalAttitude_73dnt5zb_  _knowledge_79n2fh4b risk_perception_79n2fh4t threat_susceptibility_79n2fh4s_ | 0 |  |  | PLDHQ |  |  | LB |  |  |
| Degeest et al. | _Epidemiology and risk factors for tinnitus after leisure noise exposure in Flemish young adults_ | 2017 | BE | 517 | M=40.6%  F=59.4% | M= 22.8  SD=3.5 | U  _(>50%)_ | Leisure  ‘Noisy environment’  Work | _attitude_soundculture_7c082lmy attitude_noise_7c08258d_  _autonomy_conditionPresence_73dnt5zr_  _behaviour_79n2w1bj_  _capacity_73dnt602 intention_73dnt604_  _instrumentalAttitude_73dnt5zb_  _perceivedNorms_73dnt5zq threat_susceptibility_79n2fh4s threat_severity_79n2fh4r_ | HCM (F)  HBM (R)  TPB (R) | X | X |  | X |  | 1) Behavior:  HP use during several leisure time and work activities  (yes/no)  _(for the associations the authors used HP use during each person’s highest noise exposure activity)_ | X |  |
| Degeest et al. | _The testretest reliability of questionnaires regarding attitudes and beliefs toward noise, hearing loss, and hearing protector devices in young adults_ | 2018 | BE | 43 | M=30.2%  F=69.8% | M=24.6 SD=3.0 | U | Leisure  ‘Noisy environment’  Work | _attitude_noise_7c08258d_  _attitude_soundculture_7c082lmy autonomy_conditionPresence_73dnt5zr_  _behaviour_79n2w1bj_  _capacity_73dnt602_  _instrumentalAttitude_73dnt5zb intention_73dnt604_  _perceivedNorms_73dnt5zq_  _threat_severity_79n2fh4r threat_susceptibility_79n2fh4s_ | HCM (R)  HBM (R)  TPB (R) | X | X |  | X |  | 1) Behavior:  HP use during several leisure and work activities  (yes/no)  _(for the associations the authors used HP use during each person’s highest noise exposure activity)_  2) Behavior:  HP use (*ever used*)  (yes/no)  3) Intention to wear HP in loud environments. (5-point scale) | X |  |
| DelGiacco, A. M., Serpanos, Y. C., & Gunderson, E. | _Education and Knowledge of Noise Exposure, Hearing Loss, and Hearing Conservation in College Students_ | 2015 | US | 329 | M=26.0%  F=73.0%  0=1.0% | M=21.0  Min.=18.0  Max.=57.0 | U | Leisure  ‘Noisy environment’ | _behaviour_79n2w1bj_  _knowledge_79n2fh4b threat_susceptibility_79n2fh4s_ | 0 |  |  |  | X |  | 1) Behavior:  HP use (setting not specified) (5-point scale) |  |  |
| Dell, S. M., & Holmes, A. E. | _The effect of a hearing conservation program on adolescents' attitudes towards noise._ | 2012 | US | 64 | M=50.0%  F=50.0% | M=13.0  Min.=12.0  Max.=14.0 | S | Leisure | _attitude_noise_7c08258d_  _attitude_soundculture_7c082lmy_ | HBM (F)   TRA (R) | X |  |  |  |  | NC |  |  |
| Diviani et al. | _Awareness, attitudes, and beliefs about music-induced hearing loss: Towards the development of a health communication strategy to promote safe listening._ | 2019 | CH | 1019 | M=45.6%  F=54.1% | M=28.1  SD=4.4 |  | Leisure | _autonomy_conditionPresence_73dnt5zr_  _instrumentalAttitude_73dnt5zb_  _knowledge_79n2fh4b_  _perceivedBehavioralControl_73dnt603_  _threat_susceptibility_79n2fh4s threat_severity_79n2fh4r_ | TTM (F)  HBM (F) |  |  | LHQ |  |  | 1) LB  2) Proxy of HPB: 'Readiness' to engage in HPB  _(classification into Precontemplation, Contemplation, or Action group based on 12 statements with scores on 5-point scale)_ |  |  |
| EenVandaag | _3Vraagt: Onderzoek Gehoorbescherming_ | 2018 | NL | 1656 | Not reported _(The sample is said to be representative for Dutch citizens 16 - 34 years of age)_ | Min.=16.0  Max.=34.0 | C | Leisure | _attitude_soundculture_7c082lmy_  _behaviour_79n2w1bj_  _locus_of_control_7ddfj4wn_ | 0 |  |  |  | X |  | 1) Behavior:  HP use *(‘ever used’*) at music festival (3-point scale)  2) Behavior:  HP use *(‘ever used’*) at pop concert (3-point scale)  3) Behavior:  HP use *(‘ever used’*) at dance party (3-point scale) |  | X |
| GGD Brabant Zuidoost | _Gehoorschade en het gebruik van oordoppen_ | 2017 | NL | 675 | M= 27.4%  F= 72.6% | 16-20 yrs.: 31.0%  21-36 yrs.: 69.0% |  | Leisure | _attitude_73dnt5zc_  _autonomy_conditionPresence_73dnt5zr_  _behaviour_79n2w1bj injunctiveNorms_73dnt5zj descriptiveNorms_73dnt5zp_ | 0 |  |  |  | X |  | 1) Behavior:  HP possession (yes/no)  2) Behavior:  HP use when the music is very loud (4-point scale) |  | X |
| Gilles et al. | _Prevalence of leisure noise-induced tinnitus and the attitude toward noise in university students_ | 2012 | BE | 145 | M=30.3%  F=69.7% | M=20.8  SD=1.5 | U  _(medical students)_ | Leisure  ‘Noisy environment’ | _attitude_noise_7c08258d_  _attitude_soundculture_7c082lmy behaviour_79n2w1bj intention_73dnt604_  _knowledge_79n2fh4b perceivedBehavioralControl_73dnt603 perceivedNorms_73dnt5zq_ | TPB (R) | X | X  _(The 'Influence' and 'Knowledge' items of the scale appear to derive from the BAHPHL)_ |  | X |  | 1) Behavior:  HP use in noisy environments. (5-point scale, converted to dichotomous score)  _(Item used for measurement is from BAHPHL intention subscale. In written communication author tells that only ‘totally agree’ counted as a ‘yes’)_  2) Intentions to wear HP in the future (no setting specified) (5-point scale) |  |  |
| Gilles et al. | _Epidemiology of noise-induced tinnitus and the attitudes and beliefs towards noise and hearing protection in adolescents_ | 2013 | BE | 3892 | M=47.0%  F=53.0% | M=16.6 SD=1.3 | S | Leisure  ‘Noisy environment’ | _attitude_soundculture_7c082lmy attitude_noise_7c08258d autonomy_conditionPresence_73dnt5zr_  _behaviour_79n2w1bj capacity_73dnt602 intention_73dnt604 instrumentalAttitude_73dnt5zb threat_susceptibility_79n2fh4s threat_severity_79n2fh4r perceivedNorms_73dnt5zq_ | TPB (R) | X | X |  | X |  | 1) Behavior:  HP use in noisy environments. (5-point scale, converted to dichotomous score)  _(Item used for measurement is from BAHPHL intention subscale. In written communication author tells that only ‘totally agree’ counted as a ‘yes’)_ |  |  |
| Gilles, A., & Van de Heyning, P. | _Effectiveness of a preventive campaign for noise-induced hearing damage in adolescents_ | 2014 | BE | 547 | Not reported | M= 16.8 SD=0.8 | S | Leisure  ‘Noisy environment’ | _attitude_noise_7c08258d_  _attitude_soundculture_7c082lmy autonomy_conditionPresence_73dnt5zr_  _behaviour_79n2w1bj_  _capacity_73dnt602_  _instrumentalAttitude_73dnt5zb_  _intention_73dnt604_  _threat_severity_79n2fh4r_  _threat_susceptibility_79n2fh4s perceivedNorms_73dnt5zq_ | TPB (F) | X | X |  | X |  | 1) Behavior:  HP use in noisy environments. (5-point scale, converted to dichotomous score by authors)  _(Item used for measurement is from BAHPHL intention subscale. In written communication author tells that only ‘totally agree’ counted as a ‘yes’)_ |  |  |
| Gilles et al. | _A little bit less would be great: Adolescents opinion towards music levels_ | 2014 | BE | 790 | M=36.6%  F=63.4% | M=19.2 SD=2.1 | U  S  C | Leisure | _autonomy_belief_73dnt5zt awareness_79n2w1bj_  _behaviour_79n2w1bj  risk_perception_79n2fh4t_ | 0 |  |  |  | X |  | 1) Behavior:  HP use in discotheques/ music venues  (yes/no)  2) Behavior:  HP use at music venues (with distinction between indoor and outdoor venues)  (5-point scale) |  |  |
| Gilliver et al. | _Music to whose ears? The effect of social norms on young people’s risk perceptions of hearing damage resulting from their music listening behaviour_ | 2012 | AU | 486 | M=52.0%  F=48.0% | M=16.3 SD=1.3 | S  U | Leisure | _behaviour_79n2w1bj referentBehavior_73dnt5zk risk_perception_79n2fh4t_ | TPB (R) |  |  |  |  |  | LB |  |  |
| Gilliver, M., Beach, E. F., & Williams, W. | _Noise with attitude: influences on young people's decisions to protect their hearing._ | 2013 | AU | 1000 | M=43.0%  F=57.0% | 18-24 yrs.: 34.0%  25-29 yrs.: 34.0%  30-35 yrs.: 32.0% |  | Leisure | _attitude_73dnt5zc behaviour_79n2w1bj_  _knowledge_79n2fh4b risk_perception_79n2fh4t threat_susceptibility_79n2fh4s_ | Stage Model (F) _(created by authors)_ |  |  |  | X | X | 1) Behavior: HPB in general ('Do you take steps to protect your hearing?') (yes/no)  2) Behavior:  Avoiding and/or limiting exposure to loud noise and music _(classification into Dismissive, Unconcerned, Concerned, or Active group based on choice of one of four statements that best describes HPB of participant)_ | X |  |
| Gilliver, M., Beach, E. F., & Williams, W. | _Changing beliefs about leisure noise: Using health promotion models to investigate young people's engagement with, and attitudes towards, hearing health_ | 2015 | AU | 1196 | M=24.0%  F=76.0% | M=28.0 SD=4.7 |  | Leisure | _autonomy_conditionPresence_73dnt5zr_  _behaviour_79n2w1bj instrumentalAttitude_73dnt5zb_ _threat_severity_79n2fh4r threat_susceptibility_79n2fh4s_ | HBM (F)  TTM (F) |  |  |  | X |  | 1) Behavior: Used HP at entertainment venues (in past 6 mo.) (yes/no)  2) Behavior:  Reduced exposure to noise at entertainment venues (in past 6 months) (yes/no) | X |  |
| Gorter, A.F. | _Gehoorschade als gevolg van harde muziek: risicogedrag en misconcepties onder uitgaanspubliek_ | 2012 | NL | 130000 | Not reported | Min.=18.0  Max.=35.0 | C | Leisure | _attitude_soundculture_7c082lmy autonomy_conditionPresence_73dnt5zr_  _behaviour_79n2w1bj_  _knowledge_79n2fh4b_  _locus_of_control_7ddfj4wn_  _risk_perception_79n2fh4t_  _threat_severity_79n2fh4r threat_susceptibility_79n2fh4s_ | 0 |  |  |  | X |  | 1) Behavior: HP worn at music venue that was just visited (yes/no) |  | X |
| Griest, S. E., Folmer, R. L., & Martin, W. H. | _Effectiveness of 'dangerous decibels,' a school-based hearing loss prevention program_ | 2007 | US | 550 | M=51.0%  F=49.0% | Not reported  _(US 7th grade students, i.e. 12-13 years of age)_ | S | Leisure  ‘Noisy environment’ | _intention_73dnt604_  _knowledge_79n2fh4b threat_severity_79n2fh4r threat_susceptibility_79n2fh4s_ | TRA (F) |  |  |  | X |  | 1) Behavior:  HP use when around loud sounds (3-point scale)  2) Intention to wear HP when at a loud concert (3-point scale) |  |  |
| Gupta et al. | _Assessment of Knowledge of Harmful Effects and Exposure to Recreational Music in College Students of Delhi: A Cross Sectional Exploratory Study_ | 2014 | IN | 940 | Not reported | M=20.0 SD=2.0 | U | ‘Noisy environment’ | _behaviour_79n2w1bj knowledge_79n2fh4b_ | 0 |  |  |  | X |  | 1) Behavior:  HP use during “noisy gatherings" (yes/no) |  |  |
| Herrera et al. | _Amplified music with headphones and its implications on hearing health in teens._ | 2016 | BR | 131 | M=55.0%  F=45.0% | Min.=14.0  Max.=18.0 | S | Leisure | _threat_susceptibility_79n2fh4s_ | 0 |  |  |  |  |  | LB |  |  |
| Hickson et al. | _Australian adolescent attitudes toward noise and the use of hearing protection_ | 2007 | AU | 287 | M=47.7%  F=52.3% | 13 - 15 yrs.: 34.1%  16 - 19 yrs.: 65.9% | S  U | Leisure  Work | _attitude_noise_7c08258d attitude_soundculture_7c082lmybehaviour_79n2w1bj_ | 0 | X |  | AHH | X |  | 1) Behavior:  HP use during 14 noisy activities, (3-point scale, later dichotomized by the authors)  _(HP use overall, and HP use for 7 activities extracted)_ | X |  |
| Holmes et al. | _Perceived hearing status and attitudes toward noise in young adults_ | 2007 | US | 245 | M=46.9%  F=53.1% | M=20.3 SD=2.2 | U | Leisure  Work | _attitude_noise_7c08258d attitude_soundculture_7c082lmy behaviour_79n2w1bj_ | 0 | X |  | AHH | X |  | 1) Behavior:  HP use during 11 noisy activities, (3-point scale)  _(HP use for 10 activities extracted)_ |  |  |
| Hoover, A., & Krishnamurti, S. | _Survey of college students' MP3 listening: Habits, safety issues, attitudes, and education._ | 2010 | US | 428 | M=32.7%  F=60.0%  NR=7.3% | M= 20.9  Min.=19.7  Max.=24.3 | U | Leisure | _behaviour_79n2w1bj threat_susceptibility_79n2fh4s motivation_79n2fh4q_ | 0 |  |  |  |  | X | 1) Behavior:  Use of volume limiter on PLD (3-point scale)  2) HPB proxy (x3):  a. Motivation/ willingness to decrease listening time, b. turndown volume, and c. buy special earphones for ear protection |  |  |
| Hoppenbrouwers et al. | _Attituden en zelf-gerapporteerde lawaaiblootstelling en gehoorbescherming bij 12-jarige jongeren in Vlaanderen_ | 2018 | BE | 1443 | M=45.1%  F=54.9% | M=12.8 SD=0.3 | S | Leisure  ‘Noisy environment’ | _autonomy_conditionPresence_73dnt5zr_  _behaviour_79n2w1bj instrAttitude_belief_73dnt5z8 referentApproval_73dnt5zd referentBehavior_73dnt5zk threat_severity_79n2fh4r threat_susceptibility_79n2fh4s_ | 0 | X  _(partly)_ | X  _(partly)_ |  | X |  | 1) Behavior:  Use of HP (*‘ever used’*), in any setting (yes/no) |  |  |
| Hutchinson Marron et al. | _College students' personal listening device usage and knowledge._ | 2015 | US | 180 | M=28.3%  F=71.7% | M=19.8 Min.=17.0  Max.=25.0 | U | Leisure | _risk_perception_79n2fh4t_ | 0 |  |  |  |  |  | NC  _(Limit daily PLD use “if you would know to be listening at harmful levels”)_ |  |  |
| Johnson et al. | _British university students' attitudes towards noise-induced hearing loss caused by nightclub attendance._ | 2014 | UK | 357 | M=39.8%  F=60.2% | M=21.0 SD=1.8 | U _(>50% medical students)_ | Leisure | _inflEnvironment_79n2r0sy knowledge_79n2fh4b motivation_79n2fh4q threat_susceptibility_79n2fh4s_ | 0 |  |  |  |  |  | NC  _(Motivation to adjust nightclub attendance 'if you were told that noise levels in nightclubs could lead to permanent hearing loss’)_ |  |  |
| Keppler, H., Dhooge, I., & Vinck, B. | _Hearing in young adults, part I: the effects of attitudes and beliefs toward noise, hearing loss and hearing protector devices_ | 2015 | BE | 163 | M=22.1%  F=77.9% | M=21.2  SD=2.9 |  | Leisure  ‘Noisy environment’ | _attitude_noise_7c08258d attitude_soundculture_7c082lmy autonomy_conditionPresence_73dnt5zr behaviour_79n2w1bj capacity_73dnt602_  _instrumentalAttitude_73dnt5zb_  _intention_73dnt604_  _perceivedNorms_73dnt5zq threat_susceptibility_79n2fh4s threat_severity_79n2fh4r_ | HCM (F)  HBM (R)  TPB (R) | X | X |  | X |  | 1) Behavior:  HP use *(‘ever used’*) in any setting (yes/no)  2) Intention to wear HP in loud environments (5-point scale) | X |  |
| Keppler et al. | _The effects of a hearing education program on recreational noise exposure, attitudes and beliefs toward noise, hearing loss, and hearing protector devices in young adults_ | 2015 | BE | 78 | M=12.8%  F=87.2% | M=21.0 SD=2.8 |  | Leisure  ‘Noisy environment’ | _attitude_soundculture_7c082lmy attitude_noise_7c08258d_  _autonomy_conditionPresence_73dnt5zr_  _behaviour_79n2w1bj_  _capacity_73dnt602 intention_73dnt604 instrumentalAttitude_73dnt5zb perceivedNorms_73dnt5zq threat_susceptibility_79n2fh4s threat_severity_79n2fh4r_ | HBM (R)  TPB (R)  TTM (R) | X | X |  | X |  | 1) Behavior:  HP use *(‘ever used’*) in any setting (yes/no)  2) Intention to wear HP in loud environments (5-point scale) | X |  |
| Khan et al. | _Efficacy of technology-based interventions to increase the use of hearing protections among adolescent farmworkers._ | 2018 | US | 50 | M=72.0%  F=28.0% | M=17.3 SD=0.8 | U  W _(young farmers)_ | Work | _behaviour_79n2w1bj_  _capacity_73dnt602 instrumentalAttitude_73dnt5zb knowledge_79n2fh4b risk_perception_79n2fh4t_ | HBM (F)  _(The HBQ is based on the HBM)_ |  |  | HBQ | X |  | 1) Behavior:  HP use during farm tasks (0 – 100% time of different tasks)  _(3 measures: a. high noise tasks, b. low noise tasks, and c. overall HP use for all noisy farm tasks)_ | X |  |
| de Lacerda, Soares, Gonçalves & Lopes | _Educational workshops as a strategy to promote hearing heath in adolescents: an exploratory study_ | 2013 | BR | 91 | M=59.0%  F=41.0% | <15 yrs.: 11.0%  15-19 yrs.: 89.0% | S | Leisure | _attitude_noise_7c08258d attitude_soundculture_7c082lmy  (For the first factor of the YANS “attitudes towards noise associated with elements of youth culture” [= 'attitude_soundculture_7c082lmy] '_ *_not_* _the Brazilian version but the original version with 8 items is extracted]_ | 0 | X  _(Brazilian version)_ |  |  |  |  | NC |  |  |
| de Lijster, G. P. A., & van der Ploeg, C. P. B. | _Bevordering van het gebruik van gehoorbescherming in de praktijk van het uitgaansleven in Zoeterwoude_ | 2015 | NL | 91 | M=57.0%  F=43.0% | M=17.7  15 yrs.: 13%  16 yrs.: 32%  17 - 27 yrs.: 55% | S | Leisure | _behaviour_79n2w1bj_  _locus_of_control_7ddfj4wn risk_perception_79n2fh4t_ | 0 |  |  |  | X |  | 1) Behavior:  HP use when at club or concert (3-point scale) |  | X |
| Lee, D., & Han, W. | _Noise levels at baseball stadiums and the spectators attitude to noise_ | 2019 | KR | 344 | M=52.4%  F=47.6% | M=29.6 Min.=13.0  Max.=65.0 |  | Leisure | _descrNorms_belief_73dnt5zm_  _knowledge_79n2fh4b_  _motivation_79n2fh4q motivationToComply_73dnt5zf risk_perception_79n2fh4t_ | 0 |  |  |  |  |  | NC  _(Motivation to wear HP ''if your ears don’t feel good due to loud cheering and shouting noise”)_ |  |  |
| Lee, H.-J., & Jeong, I. S. | _Personal Listening Device Use Habits, Listening Belief, and Perceived Change in Hearing Among Adolescents_ | 2021 | KR | 416 | M=48.8%  F=51.2% | Not reported  _(Participants were Middle and High school Students)_ | S | Leisure | _autonomy_conditionPresence_73dnt5zr_  _instrumentalAttitude_73dnt5zb threat_susceptibility_79n2fh4s threat_severity_79n2fh4r perceivedBehavioralControl_73dnt603_ | HBM (F) |  |  | LHQ |  |  | LB |  |  |
| de Lourdes Quintanilla-Dieck, M., Artunduaga, M. A., & Eavey, R. D. | _Intentional exposure to loud music: the second MTV.com survey reveals an opportunity to educate._ | 2009 | US | 2500 | M=31.0%  F=69.0% | M=21.7  Min.=9.0  Max.=31.0 |  | Leisure | _awareness_79n2w1bj behaviour_79n2w1bj_  _descrNorms_belief_73dnt5zm intention_73dnt604_  _motivationToComply_73dnt5zf risk_perception_79n2fh4t threat_susceptibility_79n2fh4s_ | 0 |  |  |  | X | X | 1) Behavior:  HP use at a concert or a loud event' (3-point scale, converted into dichotomous score by authors)  2) Intention to wear HP at a concert or club (4-point scale, converted into dichotomous score by authors)  3) Behavior:  Keep a moderate volume level on personal music players (3-point scale) |  |  |
| Martens, M., Perenboom, R., & van der Ploeg, C. | _Risicogroep analyse onder jongeren ten aanzien van blootstelling aan hard geluid_ | 2006 | NL | 505 | M=59.0%  F=41.0% | M=13.1 Min.=10.0  Max.=23.0 | S  U | Leisure | _attitude_73dnt5zc_  _attitude_soundculture_7c082lmy_  _behaviour_79n2w1bj intention_73dnt604 inflEnvironment_79n2r0sy_ | 0 |  |  |  | X |  | 1) Behavior:  HP use when music is loud (3-point scale) |  | X |
| Matei et al. | _Health education for musicians_ | 2018 | UK | 124 | M=45.7%  F=50.6%  NR=3.7% | M=19.0  SD=1.3 | M  U  W | Work | _autonomy_belief_73dnt5zt_  _behaviour_79n2w1bj_ | 0 |  |  |  | X |  | 1) Behavior:  HP use while making music (measured for 4 different conditions)  (5-point scale, converted into dichotomous score by authors) |  |  |
| Nielsen, L. B., Beach, E., & Gilliver, M. | _Clubbers’ attitude toward earplugs: better with use_ | 2014 | AU | 51 | M=72.5%  F=27.5% | M=27.1 Min.=20.0  Max.=39.0 | C | Leisure | _autonomy_belief_73dnt5zt_ | 0 |  |  |  |  |  | NC  _(HP was provided by researchers as part of a trial)_ |  |  |
| Olson et al. | _Hearing health in college instrumental musicians and prevention of hearing loss_ | 2016 | US | 90 | Not reported | Min.=18.0  Max.=30.0 | M  U | Leisure | _autonomy_conditionPower_73dnt5zs_  _behaviour_79n2w1bj motivation_79n2fh4q_ | HBM (R) |  |  |  | X |  | 1) Behavior:  HP use while making music (separately measured for rehearsal and performance)  (yes/no) |  |  |
| Peters, G., & Noijen, J. | _Party Panel 17.1._ | 2019 | NL | 1345 | M=48.1%  F=51.7%  O=0.2% | M=25.3  SD=9.3 | C | Leisure | _attitude_73dnt5zc behaviour_79n2w1bj_  _habit_79n2r0sz intention_73dnt604 perceivedNorms_73dnt5zq perceivedBehavioralControl_73dnt603_ | RAA (F) |  |  |  | X |  | 1) Behavior:  Carrying HP to music venue (7-point scale)  2) Behavior:  Wearing HP at music venue  (7-point scale)  _(Participants answered questions about either carrying, wearing, or buying HP. The latter was not extracted for the review)_  3) Intention to carry HP to music venue  (7-point scale)  4) Intention to wear HP at music venue  (7-point scale)  5) Behavior: 'Left venue because music was too loud' (yes/no)  _(The latter variable is only part of the univariate results)_ | X | X |
| Portnuff, C. D., Fligor, B. J., & Arehart, K. H. | _Teenage use of portable listening devices: A hazard to hearing?_ | 2011 | US | 29 | M=41.4%  F=58.6% | M=14.4 Min.=13.0  Max.=17.0 | S | Leisure | _autonomy_conditionPresence_73dnt5zr_  _behaviour_79n2w1bj instrumentalAttitude_73dnt5zb_  _threat_severity_79n2fh4r_  _threat_susceptibility_79n2fh4s perceivedBehavioralControl_73dnt603_ | HBM (F) |  |  | LHQ  _Laboratory_ |  |  | LB |  |  |
| Portnuff, C.D.F. | _Music-Induced Hearing Loss from Portable Listening Devices: Evaluating the Factors That Influence Risk Behaviors_ | 2011 | US | 52 | M=40.4%  F=59.6% | M=25.0 Min.=18.0  Max.=29.0 |  | Leisure | _autonomy_conditionPresence_73dnt5zr_  _behaviour_79n2w1bj_  _instrumentalAttitude_73dnt5zb_  _threat_susceptibility_79n2fh4s threat_severity_79n2fh4r perceivedBehavioralControl_73dnt603_ | HBM (F) |  |  | LHQ  _Laboratory_  _Datalogging_ |  |  | LB |  | X _(thesis)_ |
| Rawool, V. W., & Colligon-Wayne, L. A. | _Auditory lifestyles and beliefs related to hearing loss among college students in the USA._ | 2008 | US | 238 | M=16.8%  F=83.2% | Not reported  _(All students are US first year college students, i.e. 18-19 years of age)_ | U | Leisure  Work | _autonomy_conditionPresence_73dnt5zr_  _behaviour_79n2w1bj threat_susceptibility_79n2fh4s_ | HBM (F) |  |  |  | X |  | 1) Behavior:  HP use at work (yes/no)  2) Behavior:  *Not* wearing HP when using noisy tools (5-point scale) |  |  |
| Reddy et al. | _Attitudes to noise and behaviour towards hearing protection among Pasifika university students in New Zealand_ | 2021 | NZ | 96 | M=17.7%  F=82.3% | M=23.1  SD=4.5 | U | Leisure  ‘Noisy environment’ | _attitude_noise_7c08258d_  _attitude_soundculture_7c082lmy autonomy_conditionPresence_73dnt5zr_  _capacity_73dnt602 intention_73dnt604_  _instrumentalAttitude_73dnt5zb threat_severity_79n2fh4r threat_susceptibility_79n2fh4s perceivedNorms_73dnt5zq_ | 0 | X | X |  | X |  | 1) Intention to wear HP in loud environments (5-point scale) | X |  |
| Reiness, M., Daugaard, C., & Nielsen, P. | _Attitudes, rewards, and listening-habits in Danish youth_ | 2013 | DK | 1828 | M=34.0%  F=66.0% | M=17.5  Min.=16.0  Max.=19.0 | S  _(>50%)_ | Leisure | _behaviour_79n2w1bj_  _cues_79n2w1bj_  _descrNorms_belief_73dnt5zm_  _injunctiveNorms_73dnt5zj_  _knowledge_79n2fh4b motivationToComply_73dnt5zf_  _risk_perception_79n2fh4t threat_susceptibility_79n2fh4s_ | Blesser and Salter (R) |  |  |  |  | X | 1) Behavior:  PLD safe listening volume choice.  _(“Protect hearing” as reason to refrain from listening at higher sound volume on PLD (one of multiple choice options))_ |  | X _(conference presentation)_ |
| Serpanos, Y. C., Berg, A. L., & Renne, B. | _Influence of hearing risk information on the motivation and modification of personal listening device use_ | 2016 | US | 523 | M=31.2%  F=66.7%  O=1.0%  NR=1.1% | >70% 17-20 years of age  _(frequencies for age groups reported)_ | U | Leisure | _behaviour_79n2w1bj_  _knowledge_79n2fh4b_ | HBM (R)  Blesser and Salter (R)  TPB/RAA (R)  PMT (R) |  |  |  |  | X | 1) Behavior:  PLD safe sound volume to protect hearing (5-point scale)  _("I turn down the volume (at or less than half setting) when listening under earphones to protect my hearing")_ |  |  |
| Silva, E. D. D., Scharlach, R. C., & Teixeira, J. A. D. M. | _Use of digital audio players by high school students: Measurement of use intensity and usage habits_ | 2018 | BR | 57 | M=79.0%  F=21.0% | M=16.7  SD=0.6 | S | Leisure | _behaviour_79n2w1bj_  _risk_perception_79n2fh4t_ | 0 |  |  |  | X |  | 1) Behavior:  HP use, setting not specified (yes/no) | X |  |
| Steen, L. | _Sound Efficacy? Een formatieve en summatieve evaluatie van een getailorde en niet-getailorde interventie binnen de context van een gehoorschadepreventiecampagne_ | 2008 | NL | 211 | M=33.0%  F=67.0% | 16-20 yrs.: 32.2%  21-25 yrs.: 45.5%  26-30 yrs.: 22.3% | C | Leisure | _behaviour_79n2w1bj intention_73dnt604 motivation_79n2fh4q threat_susceptibility_79n2fh4s_ | PAPM (F) |  |  |  | X |  | 1) Behavior:  HPB general (“I already take measures to protect my hearing”, multiple choice option)  2) Intention:  HPB general  ('I want to take measures to protect my hearing', multiple choice option) |  | X |
| Vogel et al. | _Adolescents and MP3 players: too many risks, too few precautions._ | 2009 | NL | 1512 | M=50.5%  F=49.5% | M=14.7  SD=1.2 | S | Leisure | _behaviour_79n2w1bj_ | 0 |  |  |  |  | X | 1) Behavior:  Use noise-limiter on PLD (yes/no)  2) Behavior:  Reduced volume after a period of listening (yes/no)  3) Behavior:  Took breaks from listening (yes/no)  4) Behavior:  Heeded warnings against the risks of high-volume music (yes/no) |  |  |
| Vogel et al. | _Young People: Taking Few Precautions Against Hearing Loss in Discotheques_ | 2010 | NL | 1086 | M=47.0%  F=53.0% | M=14.8 SD=1.2 | S | Leisure | _behaviour_79n2w1bj_ | 0 |  |  |  | _(HP use already included for other study with same sample)_ |  | 1) Behavior:  Keep distance from loudspeakers when at discotheque (yes/no)  2) Behavior:  Take noise breaks when at discotheque (yes/no) |  |  |
| Vogel et al. | _Discotheques and the risk of hearing loss among youth: Risky listening behavior and its psychosocial correlates_ | 2010 | NL | 1086 | M=47.0%  F=53.0% | M=14.8  SD=1.2 | S | Leisure | _attitude_soundculture_7c082lmy_  _behaviour_79n2w1bj_  _capacity_73dnt602 habit_79n2r0sz instrAttitude_expectation_73dnt5z6_  _intention_73dnt604_  _instrumentalAttitude_73dnt5zb threat_severity_79n2fh4r threat_susceptibility_79n2fh4s_ | PMT (F) |  |  |  | X |  | 1) Intention to protect hearing during discotheque visits (5-point scale) | X |  |
| Vogel et al. | _Adolescents Risky MP3-Player Listening and Its Psychosocial Correlates_ | 2011 | NL | 1360 | M=49.9%  F=52.1% | M=14.7  SD=1.2 | S | Leisure | _attitude_soundculture_7c082lmy_  _capacity_73dnt602_  _habit_79n2r0sz_  _instrumentalAttitude_73dnt5zb_  _instrAttitude_expectation_73dnt5z6 intention_73dnt604_  _threat_severity_79n2fh4r threat_susceptibility_79n2fh4s_ | PMT (F) |  |  |  |  | X | 1) Intention not to play music too loud on PLD (5-point scale) | X |  |
| Wang et al. | _Health Education Intervention on Hearing Health Risk Behaviors in College Students_ | 2021 | CN | 830 | M=29.0%  F=71.0% | M=19.6  SD=0.8 | U _(medical students)_ | Leisure | _autonomy_belief_73dnt5zt instrumentalAttitude_73dnt5zb perceivedNorms_73dnt5zq_  _threat_susceptibility_79n2fh4s_ | HBM (F) |  |  |  |  |  | LB |  |  |
| Warner-Czyz, A. D., & Cain, S. | _Age and gender differences in children and adolescents attitudes toward noise_ | 2016 | US | 96 | M=39.6%  F=60.4% | M=14.8 SD=2.7 | S | Leisure | _attitude_soundculture_7c082lmy attitude_noise_7c08258d behaviour_79n2w1bj_ | 0 | X |  |  | X |  | 1) Behavior:  HP use during 8 high noisy activities (3-point scale)  _(The correlations are based on HP use during one high-noise activity the participant is active in)_ | X |  |
| Weichbold et al. | _Effects of a hearing protection campaign on the discotheque attendance habits of high-school students_ | 2003 | AT | 169 | M=31.9%  F=68.1% | M=16.9  Min.=15.0  Max.=19.0 | S | Leisure | _behaviour_79n2w1bj_  _NC_ | 0 |  |  |  | X |  | 1) Behavior:  HP use when attending a discotheque (4-point scale) |  |  |
| Weichbold et al. | _Can a hearing education campaign for adolescents change their music listening behavior?_ | 2007 | AT | 1757 | M=49.0%  F=51.0% | M=16.2 SD=1.3 | S | Leisure | _behaviour_79n2w1bj_  _NC_ | 0 |  |  |  | X |  | 1) Behavior:  HP use when attending a discotheque (4-point scale)  2) Behavior: Take noise breaks when at discotheque (4-point scale) |  |  |
| Welch et al. | _Educating teenagers about hearing health by training them to educate children_ | 2016 | NZ | 67 | M=20.9%  F=79.1% | M=15.3  Min.=14.0  Max.=17.0 | S | Leisure | _attitude_73dnt5zc_  _autonomy_conditionPresence_73dnt5zr_  _intention_73dnt604 instrumentalAttitude_73dnt5zb knowledge_79n2fh4b_ | TRA  *_(trough “Dangerous Decibels” program)_* |  |  | HPA-5 | X |  | 1) Intention to wear HP at a loud, noisy event (3-point scale) | X |  |
| Welch, D., Ma, E., & Reddy, R. | _Hearing-health intervention for nightclub staff_ | 2019 | NZ | 20 | M=95.0%  F=5.0% | M=27.7  SD=NR | W | Work | _attitude_73dnt5zc_  _autonomy_conditionPresence_73dnt5zr_  _behaviour_79n2w1bj_  _instrumentalAttitude_73dnt5zb knowledge_79n2fh4b_ | CAALM model (R)  COM-B model (R)  TRA  _(trough “Dangerous Decibels” program)_ |  |  | HPA-5 | X |  | 1) Behavior:  HP use when around loud sounds in nightclub (5-point scale) | X |  |
| West, E. | _Learning for Everyday Life: Students' Standpoints on Loud Sounds and Use of Hearing Protectors before and after a Teaching-Learning Intervention_ | 2012 | SE | 145  _(pupils from grade 4 are excluded and not counted here)_ | M=57.2%  F=42.8%  _(grade 4 not included)_ | Min.=12.0  Max.=14.0  _(grade 4 not included)_ | S | Leisure | _risk_perception_79n2fh4t threat_susceptibility_79n2fh4s_ | 0 |  |  |  |  |  | NC  _(Only change scores reported for HPBs, no baseline results)_ |  |  |
| Widén, S. O., & Erlandsson, S. | _The influence of socio-economic status on adolescent attitude to social noise and hearing protection._ | 2004 | SE | 1285 | M=48.2%  F=51.8% | Min.=13.0  Max.=19.0 | S | Leisure | _attitude_soundculture_7c082lmy attitude_noise_7c08258d behaviour_79n2w1bj_ | 0 | X _(18-item version)_ |  | AHH   HOL | X |  | 1) Behavior:  HP use at discos and pop concerts (yes/no) | X |  |
| Widén, S. E., Holmes, A., & Erlandsson, S. | _Reported hearing protection use in young adults from Sweden and the USA: Effects of attitude and gender_ | 2006 | SE & US | 382  _N=179 (SE)_  _N=203 (US)_ | M=31.8%  F=68.2%  (SE)  M=44.3%  F=55.7%  (US) | Min.=17.0  Max.=21.0  _(for N=382)_ | U _(US)_  S _(SE)_ | Leisure | _attitude_soundculture_7c082lmy attitude_noise_7c08258d behaviour_79n2w1bj_ | TTM (R) | X |  | HSD | X |  | 1) Behavior:  HP use at discos and pop concerts (yes/no) | X |  |
| Widén et al. | _Hearing, use of hearing protection, and attitudes towards noise among young American adults_ | 2009 | US | 258 | M=28.0%  F=72.0% | M=19.0 Min.=17.0  Max.=21.0 | S | Leisure | _attitude_noise_7c08258d attitude_soundculture_7c082lmy behaviour_79n2w1bj_ | HBM (F)  HCM (F) | X |  | AHH    HSD | X |  | 1) Behavior:  HP use during 10 noisy leisure time activities (3-points scale, converted into dichotomous scale by authors) | X  _(only for 'lawn mowing' prevalence of HP use is high enough to enable estimation of association)_ |  |
| Widén, S. E., Bohlin, M., & Johansson, I. | _Gender perspectives in psychometrics related to leisure time noise exposure and use of hearing protection_ | 2011 | SE | 543 | M=50.3%  F=49.7% | Min.=16.0  Max.=20.0 | S | Leisure | _attitude_73dnt5zc_  _attitude_soundculture_7c082lmy_  _behaviour_79n2w1bj  prototype_79n2fh4t risk_perception_79n2fh4t_  _threat_susceptibility_79n2fh4s_ | 0 | X  _(YANS-R, 11 items)_ |  | AHH-R   NAIS  PSN  RPI | X |  | 1) Behavior:  HP use at discos and pop concerts  (0 - 100% of time) | X |  |
| Widén, S. E. | _A suggested model for decision-making regarding hearing conservation: Towards a systems theory approach_ | 2013 | SE | 242 | M=54.5%  F=44.6%  NR=0.9% | M=17.0 Min.=15.0  Max.=19.0 | S | Leisure | _attitude_73dnt5zc_  _attitude_soundculture_7c082lmy autonomy_conditionPresence_73dnt5zr_  _behaviour_79n2w1bj instrumentalAttitude_73dnt5zb intention_73dnt604_ | HCM (F)   HBM (F)  TPB (F) | X  _(YANS-R, 11 items)_ |  |  | X  . |  | 1) Behavior:  HP use at pop concerts (0 - 100% of time)  2) Intention to wear HP in loud music environments (5-point scale) | X |  |
| You, S., Kwak, C., & Han, W. | _Use of Personal Listening Devices and Knowledge/Attitude for Greater Hearing Conservation in College Students: Data Analysis and Regression Model Based on 1009 Respondents_ | 2020 | KR | 1009 | M=50.1%  F=49.9% | ≤19 yrs.: 1.8%  20-24 yrs.:  66.8%  24-29 yrs.:  30.6%  ≥30 yrs.: 0.8% | U | Leisure | _awareness_79n2w1bj_  _instrumentalAttitude_73dnt5zb intention_73dnt604 knowledge_79n2fh4b risk_perception_79n2fh4t threat_susceptibility_79n2fh4s_ | 0 |  |  | PLDHQ  _(Korean version)_ |  | X | 1) Intention to use volume restriction (4-point scale) |  |  |
| Zieltjens, L. S., de Lijster, G. P. A., & van der Ploeg, C. P. B. | _Gehoorschade door harde muziek: mogelijkheden voor preventie van gehoorschade bij bezoekers Jongerencentrum Zoeterwoude_ | 2014 | NL | 95 | M=46.0%  F=54.0% | M=16.7  SD=2.8 | S | Leisure | _attitude_73dnt5zc_  _attitude_soundculture_7c082lmy_  _behaviour_79n2w1bj_  _descrNorms_belief_73dnt5zm knowledge_79n2fh4b_  _prototype_79n2fh4t threat_susceptibility_79n2fh4s_ | PAPM (F) |  |  |  | X |  | 1) Behavior:  HP use  _('Are you doing something to prevent hearing damage?', open question: % of participants that mention the use of HP)_  2) Behavior:  HPB (any)  _('Are you doing something to prevent hearing damage?', open question: % of participants that mention to engage in any HPB)_ |  | X |
| Zocoli et al. | _Brazilian young adults and noise: Attitudes, habits, and audiological characteristics_ | 2009 | BR | 245 | M=49.0%  F=51.0% | M=15.7 Min.=14.0  Max.=18.0 | S | Leisure | _attitude_noise_7c08258d_  _attitude_soundculture_7c082lmy_  _behaviour_79n2w1bj_ | 0 | X  _(Brazilian version of YANS)_ |  |  | X |  | 1) Behavior:  HPB use in night clubs or at concerts (yes/no) | _(prevalence of earplug use too low – 2.0% - to enable calculation of associations)_ |  |
| Zweet, D., & Eilering, M. | _Onderzoeksresultaten campagne I Love My Ears_ | 2018 | NL | 627  _(the total sample was larger, but only data of participants ≤ 35 yrs. was included in the present review)_ | M=43.1%  F=46.9%  _(for N=627)_ | M=26.3 SD=5.8  _(for N=627)_ | C | Leisure | _autonomy_73dnt5zx behaviour_79n2w1bj capacity_73dnt602_  _descriptiveNorms_73dnt5zp_  _experientialAttitude_73dnt5z5_  _instrumentalAttitude_73dnt5zb_  _injunctiveNorms_73dnt5zj_  _intention_73dnt604_  _knowledge_79n2fh4b_  _locus_of_control_7ddfj4wn_  _threat_severity_79n2fh4r_  _threat_susceptibility_79n2fh4s_ | 0 |  |  |  | X |  | 1) Behavior:  Bring HP to club (7-point scale)  2) Behavior:  Bring HP to concert (7-point scale)  3) Intention to wear HP at next music event (7-point scale)  4) Behavior:  Keep distance from speakers during music events (7-point scale)  5) Behavior:  Take noise breaks at music events (7-point scale) | X | X |

**Notes:**

- For each study a DCT is mentioned only once but can be extracted multiple times.
- Variables, theories and instruments, and associations are only included in the table when they are extracted (or – for variables - an influence on the data extracted is likely). In the column “PLD use” only studies are shown in which PLD use is measured as a HPB (!). Studies (only) measuring PLD listening behavior (LB) are *not* shown.
- “Hearing risk behavior” is outside the scope of the present review. Therefore, PLD use patterns in itself are not included in the review, unless a PLD use pattern is explicitly guided by hearing protective motives (i.e. a choice for a certain volume, frequency, and/or duration of PLD use in itself is not necessarily a HPB).
- The colon “group” is left blank if a sample is not dominated by one particular group or if information regarding group characteristics is missing for that study.

**Abbreviations:**

1. **General:**

HPB = Hearing Protection Behavior

HP = Hearing Protection

HI = Hearing Impairment

M = mean

Mdn = median

Min.= minimum

Max.= maximum

O = other

NR = no response/not reported

PLD = Personal Listening Device

Due to two reasons no HPB or psychological construct was included in the review for a number of studies:

1. NC = Non Comparable. Results are not included in the review if measured in a way that makes comparison or aggregation impossible.
2. LB = Listening Behavior. Behaviors can not be considered HPBs and can, thus, not be included in the review, if the motives to choose a certain sound volume, duration, and/or frequency are unknown.
3. **Samples:**

U = University or college students

S = School students

M = Musicians or DJs

C = Clubbers/music venue visitors

W = Workers

*N.B.* The “group” field is left blank if the sample is not dominated by one particular group or if information regarding group characteristics is missing.

1. **Instruments:**

AHH = Adolescents’ Habits and Use of Hearing Protection scale (Olsen Widén, 2004a).

BAHPHL = The Beliefs About Hearing Protection and Hearing Loss scale (24 items, adapted Dutch version) (Keppler, 2010).

HBQ = Hearing Beliefs Questionnaire (Saunders, Frederick, Silverman, & Papesh, 2013).

HPA-5 = Hearing Protection Assessment questionnaire - adopted for nightclub settings (Reddy, Welch, Ameratunga, & Thorne, 2014).

HOL = Hollingshead Four Factor Index of Social Status (Hollingshead, 1975).

HSD = Hearing Symptom Description scale (Olsen Widén, 2004b).

LHQ = Listening Habits Questionnaire (Portnuff, 2011).

NAIS = Norms and ideals scale (Widén, Bohlin, & Johansson, 2011).

PLDHQ = Personal Listening Device and Hearing Questionnaire (Danhauer et al., 2009).

PSN = Perceived susceptibility to noise scale (Widén et al., 2011).

RPI = Risk perception inventory (Widén et al., 2011).

RCQ = Readiness to Change Questionnaire (RCQ) (Rollnick, Heather, Gold, & Hall, 1992).

YANS = Youth Attitudes to Noise Scale (adapted Dutch version) (Keppler, 2010).

1. **Theories and models:**

Blesser and Salter = The unexamined rewards for excessive loudness (Blesser & Salter, 2008).

CAALM model = The Conditioning, Adaptation and Acculturation to Loud Music model (Welch & Fremaux, 2017)

COM-B model = Capability, Opportunity, Motivation – Behaviour model (Michie, van Stralen, & West, 2011)

HBM = Health Belief Model (Rosenstock, 1974).

HCM = Hearing Conservation Model (Model of decision-making regarding hearing conservation) (Widén, 2013).

PAPM = Precaution Adoption Process Model (Weinstein, Sandman, & Blalock, 2020).

PMT = Protection Motivation Theory (Rogers, 1975).

RAA = Reasoned Action Approach (Fishbein & Ajzen, 2010).

TPB = Theory of Planned Behavior (Ajzen, 1991).

TRA = Theory of Reasoned Action (Fishbein & Ajzen, 2010).

TTM = Transtheoretical Model (Stages of Change) (Prochaska & DiClemente, 2005).

R = reference; F = framework

References

Ajzen, I. (1991). The theory of planned behavior. *Organizational Behavior and Human Decision Processes, 50*(2), 179-211.

Blesser, B., & Salter, L.-R. (2008). *The unexamined rewards for excessive loudness.* Paper presented at the Communications: 9th International Congress on Noise as a Public Health Program. Retrieved from www. blesser. net/downloads/ICBEN.

Danhauer, J. L., Johnson, C. E., Byrd, A., DeGood, L., Meuel, C., Pecile, A., & Koch, L. L. (2009). Survey of college students on iPod use and hearing health. *Journal of the American Academy of Audiology, 20*(1), 5-27.

Fishbein, M., & Ajzen, I. (2010). *Predicting and changing behavior: The reasoned action approach*: Taylor & Francis.

Hollingshead, A. B. (1975). Four factor index of social status. In: New Haven, CT.

Keppler, H. (2010). *Optimization of the diagnosis of noise-induced hearing loss with otoacoustic emissions.* Ghent University,

Michie, S., van Stralen, M. M., & West, R. (2011). The behaviour change wheel: a new method for characterising and designing behaviour change interventions. *Implementation science : IS, 6*, 42-42. doi:10.1186/1748-5908-6-42

Olsen Widén, S. (2004a). Adolescent habits and use of hearing protection (AHH). In *Psychological aspects of adolescents' perceptions and habits in noisy environments. Licentiate Dissertation.*: Department of Psychology, Göteborg University.

Olsen Widén, S. (2004b). Hearing symptom description (HSD). In *Psychological aspects of adolescents' perceptions and habits in noisy environments. Licentiate Dissertation.*: Department of Psychology, Göteborg University.

Portnuff, C. D. (2011). *Music-induced hearing loss from portable listening devices: Evaluating the factors that influence risk behaviors.* University of Colorado.

Prochaska, J. O., & DiClemente, C. C. (2005). The transtheoretical approach. *Handbook of psychotherapy integration, 2*, 147-171.

Reddy, R., Welch, D., Ameratunga, S., & Thorne, P. (2014). Development of the hearing protection assessment (HPA-2) questionnaire. *Occupational Medicine, 64*(3), 198-205. doi:10.1093/occmed/kqt178

Rogers, R. W. (1975). A protection motivation theory of fear appeals and attitude change. *The journal of psychology, 91*(1), 93-114.

Rollnick, S., Heather, N., Gold, R., & Hall, W. (1992). Development of a short 'readiness to change' questionnaire for use in brief, opportunistic interventions among excessive drinkers. *British journal of addiction, 87*, 743-754. doi:10.1111/j.1360-0443.1992.tb02720.x

Rosenstock, I. M. (1974). Historical Origins of the Health Belief Model. *Health Education Monographs, 2*(4), 328-335. doi:10.1177/109019817400200403

Saunders, G. H., Frederick, M. T., Silverman, S., & Papesh, M. (2013). Application of the health belief model: Development of the hearing beliefs questionnaire (HBQ) and its associations with hearing health behaviors. *International Journal of Audiology, 52*(8), 558-567.

Weinstein, N. D., Sandman, P. M., & Blalock, S. J. (2020). The Precaution Adoption Process Model. In *The Wiley Encyclopedia of Health Psychology* (pp. 495-506).

Welch, D., & Fremaux, G. (2017). Understanding Why People Enjoy Loud Sound. *Seminars in hearing, 38*(4), 348-358. doi:10.1055/s-0037-1606328

Widén, S. E. (2013). A suggested model for decision-making regarding hearing conservation: Towards a systems theory approach. *International Journal of Audiology, 52*(1), 57-64.

Widén, S. E., Bohlin, M., & Johansson, I. (2011). Gender perspectives in psychometrics related to leisure time noise exposure and use of hearing protection. *Noise and Health, 13*(55), 407.
